# Towards Bacterial Resistance *via* the Membrane Strategy: Enzymatic, Biophysical and Biomimetic Studies of the Lipid *cis*‐*trans* Isomerase of *Pseudomonas aeruginosa*

**DOI:** 10.1002/cbic.202400844

**Published:** 2024-11-28

**Authors:** Mickaël Mauger, Iryna Makarchuk, Yasmin Molter, Anna Sansone, Frédéric Melin, Philippe Chaignon, Philippe Schaeffer, Pierre Adam, Volker Schünemann, Petra Hellwig, Carla Ferreri, Chryssostomos Chatgilialoglu, Myriam Seemann

**Affiliations:** ^1^ Equipe Chimie Biologique et Applications Thérapeutiques, Institut de Chimie de Strasbourg UMR 7177 Université de Strasbourg, CNRS 67000 Strasbourg France; ^2^ Laboratoire de Bioélectrochimie et Spectroscopie, Chimie de la Matière Complexe UMR 7140 Université de Strasbourg, CNRS 67000 Strasbourg France; ^3^ Department of Physics University of Kaiserslautern-Landau Erwin-Schrödinger-Str. 46 67663 Kaiserslautern Germany; ^4^ Institute for Organic Synthesis and Photoreactivity National Research Council 40129 Bologna Italy; ^5^ Equipe Biogéochimie Moléculaire, Institut de Chimie de Strasbourg UMR 7177 Université de Strasbourg, CNRS 67000 Strasbourg France; ^6^ Institut Universitaire de France (IUF) France; ^7^ Center for Advanced Technologies Adam Mickiewicz University 61–614 Poznań Poland

**Keywords:** Bacterial resistance, Biophysics, Enzyme catalysis, Heme protein, Lipid *cis*-*trans* isomerase

## Abstract

The lipid *cis*‐*trans* isomerase (Cti) is a periplasmic heme‐*c* enzyme found in several bacteria including *Pseudomonas aeruginosa*, a pathogen known for causing nosocomial infections. This metalloenzyme catalyzes the *cis*‐*trans* isomerization of unsaturated fatty acids in order to rapidly modulate membrane fluidity in response to stresses that impede bacterial growth. As a consequence, breakthrough in the elucidation of the mechanism of this metalloenzyme might lead to new strategies to combat bacterial antibiotic resistance. We report the first comprehensive biochemical, electrochemical and spectroscopic characterization of a Cti enzyme. This has been possible by the successful purification of Cti from *P. aeruginosa* (*Pa*‐Cti) in favorable yields with enzyme activity of 0.41 μmol/min/mg when tested with palmitoleic acid. Through a synergistic approach involving enzymology, site‐directed mutagenesis, Raman spectroscopy, Mössbauer spectroscopy and electrochemistry, we identified the heme coordination and redox state, pinpointing Met163 as the sixth ligand of the Fe^II^ of heme‐*c* in *Pa*‐Cti. Significantly, the development of an innovative assay based on liposomes demonstrated for the first time that Cti catalyzes *cis*‐*trans* isomerization directly using phospholipids as substrates without the need of protein partners, answering the important question about the substrate of Cti within the bacterial membrane.

## Graphical abstract

The lipid *cis*‐*trans* isomerase (Cti), a heme‐dependent enzyme, induces a swift membrane rigidification in *Pseudomonas aeruginosa* under stress conditions that impede bacterial growth. This study provides understanding of the enzyme's heme coordination and redox state as well as some insights into the catalytic mechanism and reveals that phospholipids, alongside fatty acids, serve as substrate for this metalloenzyme.

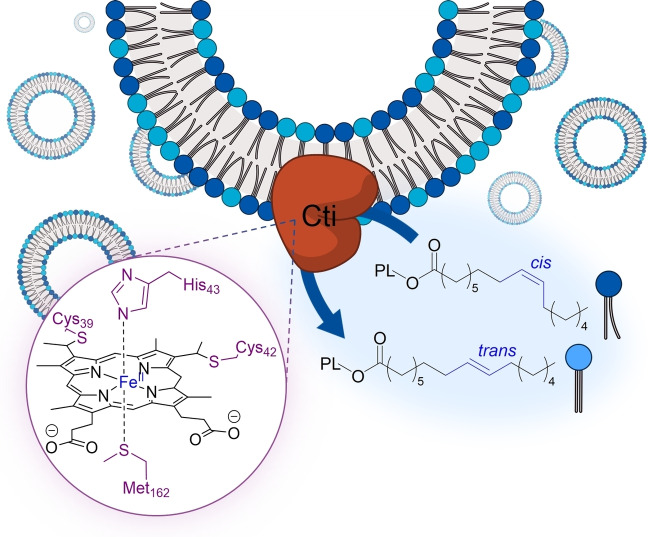

## Introduction

The widespread and repeated use of antibiotics has resulted in the emergence of bacteria resistant to these medications. Antimicrobial resistance is the cause of more than 33,000 deaths per year in Europe and could potentially become the leading cause of death globally by 2050.^[1]^ Thus, it is imperative to urgently seek new solutions to combat multi‐drug‐resistant bacteria. One source of inspiration is the investigation of specific bacterial resistance mechanisms that have not yet been used in antibiotic strategies.

In *Pseudomonas aeruginosa*, a bacterium classified as high priority by the WHO,^[2]^ a significant adaptive response involves maintaining or reducing membrane fluidity through alteration of the fatty acid composition of membrane lipids.^[3]^ This homeoviscous adaptation is triggered in *Pseudomonas* strains, as well as in *Vibrio cholerae* ‐ the agent responsible for cholera ‐, by the synthesis of *trans*‐unsaturated fatty acids (*trans*‐UFAs) achieved through the direct isomerization of the *cis* configuration of double bonds by the enzyme Cti.[^4^, ^5^, ^6^, ^7^] The conversion from *cis* to *trans* UFAs enables a swift decrease of membrane fluidity in response to adverse environmental conditions where bacterial growth is inhibited such as the presence of organic solvents, heavy metals or antibiotics acting on the membrane.[^6^, ^7^] The antibiotics nigericin and polymyxin B have been reported to induce *cis*‐*trans* isomerization of UFAs in *P. putida* S12. Moreover, *P. putida* S12 adapted to toluene, a solvent known to induce *cis*‐*trans* isomerization, exhibited over a 1000‐fold increase in survival when exposed to chloramphenicol, tetracycline or piperacillin, compared to non‐adapted bacteria.^[8]^ Consequently, Cti emerges as a promising target for the development of novel antibacterial strategies in the fight against certain antibiotic‐resistant bacteria, including *P. aeruginosa*. So far, the *cis‐trans* isomerase enzyme is not known in eukaryotic cells, having natural unsaturated lipids with only the *cis* geometry.^[9]^ Therefore, targeting the inhibition of Cti could result in an antibiotic strategy without toxicity for human cells.

*P. aeruginosa* Cti (*Pa*‐Cti) contains the CXXCH motif that was shown previously using site‐directed mutagenesis on the *P. putida* P8 orthologue to be responsible for the covalent attachment of a heme indicating that Cti is a cytochrome‐*c* type enzyme.[^10^, ^11^] Despite *in vivo* studies performed on the Cti enzyme, its molecular mechanism is not yet elucidated.[^4^, ^5^, ^6^, ^7^] In the past, only two native Cti proteins have been successfully purified: the enzyme from *P. oleovorans* GPo12 and *Pseudomonas* sp. E‐3.[^12^, ^13^] Only one study has reported the recombinant production of the Cti enzyme from *P. putida* P8 using *Escherichia coli* cells.^[10]^ The quantities of recombinant Cti obtained in this study were so limited that only a very faint UV‐Visible spectrum could be recorded. Very recently, the groups of Park and Chang were able to express and purify the recombinant Cti from *P. putida* KT2440, but still no biophysical characterization of the enzyme has been reported to date.^[14]^

Here, we present a strategy to produce Cti from the pathogenic bacterium *P. aeruginosa* in high yields, investigating also the influence of additives to ameliorate biosynthetic performance. The results enabled further structural, mechanistic, site‐specific mutation and spectroscopic studies to be carried out giving first insight into the enzymatic mechanism.

## Results and Discussion

### Production of the Periplasmic *Pa*‐Cti Holoenzyme

**Figure 1:**
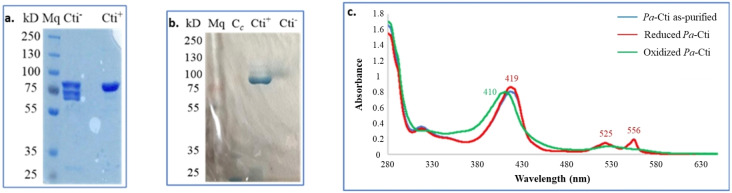
Analysis of purified *Pa*‐Cti. a. *Pa*‐Cti after purification when produced in the absence and presence of *cytochrome‐*c *maturation (ccm)* genes. 10 % SDS‐PAGE Gel. Mq: molecular weight marker; Cti^−^: purified *Pa*‐Cti expressed in absence of *ccm* genes; Cti^+^: purified *Pa*‐Cti expressed in the presence of *ccm* genes. b. Peroxidase activity of heme‐*c*. 10 % SDS‐PAGE Gel. Mq: molecular weight marker; C_c_: horse heart cytochrome‐*c*; Cti^+^: purified *Pa*‐Cti expressed in the presence of *ccm* genes; Cti^−^: purified *Pa*‐Cti expressed in absence of *ccm* genes. c. UV‐Visible absorption spectrum. 12 μM (~1 mg/mL) solution of purified *Pa*‐Cti protein (blue, partially covered by the red trace), reduced with 120 μM dithionite (red), and oxidized using 120 μM potassium ferricyanide (green). The α, β, and γ bands of the reduced form of Cti exhibit maxima at 556, 525, and 419 nm, respectively.

The covalent attachment of a heme to a polypeptide (apocytochrome‐*c*) *o*ccurs in the periplasm of *E. coli* and involves the *cytochrome‐c maturation (ccm)* machinery coded by eight genes, *ccmABCDEFGH*. However, despite the endogenous presence of *ccm* genes on the *E. coli* chromosome, they are not expressed under aerobic conditions.[^15^, ^16^] The pEC86 plasmid, which enables the constitutive expression of *E. coli ccm* genes, has proven to be a valuable tool for enhancing the maturation of type‐*c* cytochromes in *E. coli*.[^17^, ^18^, ^19^] Therefore, the gene coding *Pa*‐Cti was inserted into the pET22b vector and the corresponding plasmid was introduced in *E. coli* cells containing also the pEC86 plasmid. Plasmid pET22b allows the substitution of the *N*‐terminus native signal peptide of *Pa*‐Cti by the pelB signal peptide,^[20]^ enabling the transfer of the fused protein into the periplasmic space of Gram‐negative bacteria. Expression and purification of *Pa*‐Cti were performed under optimized conditions in order to obtain the highest apocytochrome‐*c* yields. The recovered bacterial pellet exhibited a reddish‐brown color, suggesting the presence of heme‐containing protein.^[21]^ A similar approach has been reported for *P. putida* KT2240 Cti protein.^[14]^ Differently from the published work, we recovered the protein from the periplasmic space using a modified osmotic shock^[22]^ in order to extract *Pa*‐Cti in the holoenzyme form (that is only present in the periplasm). *Pa*‐Cti was subsequently purified using Ni^II^‐NTA affinity chromatography (Figure S1a). Interestingly, we have observed that a higher amount of *Pa*‐Cti protein was obtained (2.4 instead of 1.2 mg per L of culture) when cell lysis was performed by sonication instead of osmotic shock, but the resulting *Pa*‐Cti was found to be degraded (Figure S2). Apocytochrome‐*c* proteins present in the cytoplasm are generally degraded *in vivo* due to the absence of the heme.^[23]^ Hence, the degradation bands observed on the SDS‐PAGE gel (Figure S2) could be possibly attributed to the apoform of *Pa*‐Cti that was extracted from the cytoplasm by sonication. Purified *Pa*‐Cti displayed an apparent mass of about 86 kDa (Figure S1b) on the SDS‐PAGE gel in accordance with the theoretical calculated mass of 85,978.68 Da, and size‐exclusion chromatography revealed a monomeric structure. When pEC86 vector was absent, the recovered enzyme was degraded (Figure 1a). Its addition led to the production of undegraded *Pa*‐Cti (Figure 1a) with an improved yield (1.2 mg per L of culture). Further supplementation of the bacterial cells before induction of *Pa*‐Cti with iron, which is typically the limiting factor in heme biosynthesis,^[24]^ resulted in a threefold increase in the Cti production yield.

Proteins containing heme exhibit peroxidase activity, which can be easily detected using chromogenic substrates like 3,3′,5,5′‐tetramethylbenzidine.[^25^, ^26^] Since heme is covalently bound *via* thioether linkages in type‐*c* cytochromes,[^27^, ^28^, ^29^] it remains attached to the polypeptide during SDS‐PAGE analysis and subsequent peroxidase activity detection can serve as a method to evidence the presence of the heme in the obtained *Pa*‐Cti protein. Peroxidase activity was actually observed only for recombinant *Pa*‐Cti produced in the presence of the pEC86 (Figure 1b). Additionally, the UV‐Visible spectra of *Pa*‐Cti in its oxidized and reduced forms (Figure 1c) confirmed the presence of the heme. The reduced form of *Pa*‐Cti displays three characteristic absorption bands: α band=556 nm, β band=525 nm, and γ band=419 nm. According to the UV‐Visible spectrum, purified *Pa*‐Cti appears to be in its reduced state.

The successful optimizations carried out during this study enabled the obtention of satisfying quantities of *Pa‐*Cti (3.4–4.5 mg per L of culture) suitable for further activity studies as well as deep biophysical characterizations of a Cti enzyme.

### *In vivo* Activity of *Pa*‐Cti Using *E. coli* as Host

*E. coli* naturally lacks a *cis*‐*trans* isomerase.[^4^, ^5^, ^6^, ^7^, ^30^, ^31^, ^32^] However, the introduction of a *cti* gene in *E. coli* through genetic engineering renders this bacterium capable of producing *trans*‐UFAs from some of its natural *cis*‐UFAs.[^31^, ^33^, ^34^]

*In vivo* activity of *Pa*‐Cti was directly investigated in *E. coli* BL21(DE3)[pET22b/*Pa‐cti*; pEC86]. No bacterial growth was observed when this bacterium was cultivated on Petri dishes containing 100 μM IPTG. A similar growth inhibition had been previously reported by Kondakova *et al*. for *E. coli* strains expressing the *cti* gene from *P. putida* F1 after arabinose induction.^[35]^ It was suggested that overexpression of the periplasmic enzyme might lead to a lethal blockade of *E. coli* secretion machinery. To circumvent this issue, the expression of *Pa*‐Cti was induced at a basal level by acclimating *E. coli* cells on solid media containing only 10 μM IPTG, leading to colonies that could be subsequently cultured in LB medium containing 10 μM IPTG.

In order to confirm the *in vivo* activity of *Pa*‐Cti in our construction, the membrane fatty acids of *E. coli* BL21(DE3)[pET22b/*Pa‐cti*; pEC86] were isolated and analyzed following stress induction. Stress was induced by adding octanol when the bacterial culture reached an OD_600_ of 0.6. After further growth for 2 h at 30 °C, trichloroacetic acid was immediately added to halt bacterial metabolism. The fatty acid composition of membrane lipids was determined using GC analysis of the fatty acid methyl esters (FAME) obtained after saponification of membrane phospholipids and subsequent fatty acid methylation. The basal *Pa*‐Cti expression in the cells was also monitored using the Western blot method (Figure S3). Several control experiments were conducted using *E. coli* BL21(DE3)[pET22b; pEC86], which does not overexpress *Pa‐*Cti as well as control samples consisting of non‐induced and non‐stressed, or non‐induced and stressed cells (Figure S3, Columns 1 to 6). The Western blot analysis of the different bacterial pellets confirmed that only BL21(DE3)[pET22b/*Pa‐cti*; pEC86] cells containing the *cti* gene and treated with IPTG express *Pa*‐Cti (Figure S3, Columns 7 and 8).

**Figure 2:**
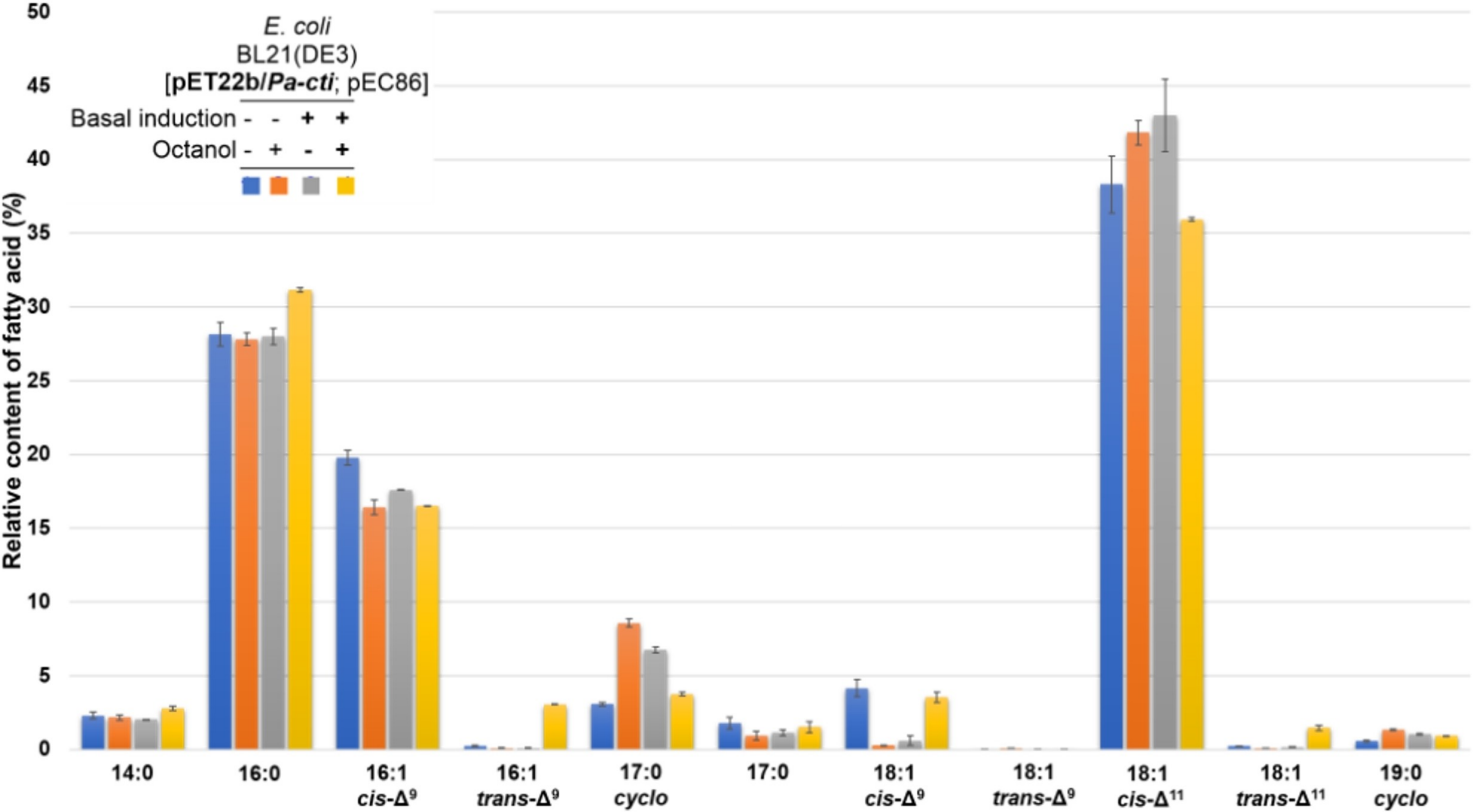
Analysis of the bacterial membrane to test for *in vivo* activity of *Pa*‐Cti. *E. coli* BL21(DE3)[pEC86] were transformed with pET22b/*Pa‐cti* plasmid ( ▪ , ▪ , ▪ , ▪ ) and cultured at 30 °C. Basal induction of *Pa*‐Cti was achieved by adding 10 μM IPTG ( ▪ , ▪ ). Stress was initiated by adding octanol to a final concentration of 2.5 mM ( ▪ ). *E. coli* uses the anaerobic pathway for fatty acid biosynthesis.[^37^, ^39^] As 18 : 1 *cis*‐Δ^9^ is produced *via* the aerobic pathway, the low amounts of 18 : 1 *cis*‐Δ^9^ most probably originated from the culture medium.

According to the literature, *E. coli* contains saturated fatty acids (SFAs;12 : 0, 14 : 0, 16 : 0, and 18 : 0), unsaturated fatty acids (UFAs; 16 : 1 *cis*‐Δ^9^ and 18 : 1 *cis*‐Δ^11^), and cyclopropane fatty acids (CFAs; 17 : 0 cyclo and 19 : 0 cyclo). Among these, palmitic acid (16 : 0), palmitoleic acid (16 : 1 *cis*‐Δ^9^), and *cis*‐vaccenic acid (18 : 1 *cis*‐Δ^11^) are the most significant components and constitute more than 90 % of the fatty acid composition in exponentially growing *E. coli* cells.[^36^, ^37^, ^38^] These fatty acids also predominate in the cell analysis (Figure 2). Importantly, only the cells in which *Pa*‐Cti expression is induced by IPTG exhibited a pronounced response to the stress generated by the addition of octanol, leading to the formation of *trans*‐isomers of 16 : 1 *cis*‐Δ^9^ and 18 : 1 *cis*‐Δ^11^ (Figure 2). Overall, as evidenced in Figure S4, the response of stressed bacteria with induced *Pa*‐Cti is characterized by a slight increase in the proportion of SFAs (from 31.5 % to 35.8 %) and a reduction in *cis*‐UFAs (from 61.5 % to 56.1 %), along with a concurrent increase in *trans*‐UFAs (from 0.35 % to 4.6 %) due to the *cis*‐*trans* isomerization of fatty acids. Non‐induced bacteria do not exhibit the formation of *trans*‐isomers in response to stress caused by octanol (Figure 2).

### *In vitro* Activity of *Pa*‐Cti Using Fatty Acids as Substrate

**Figure 3:**
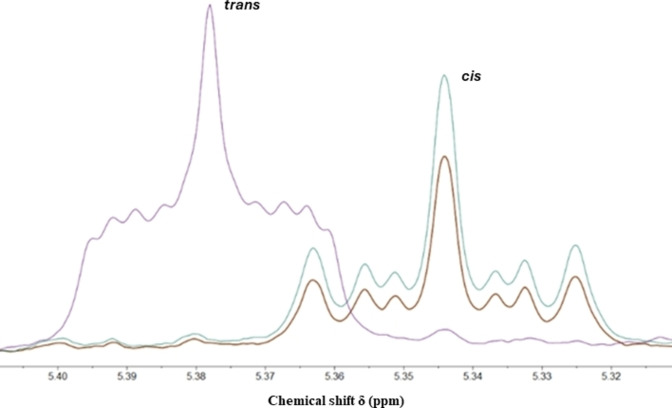
^1^H NMR spectra (300 MHz) of the FAME obtained after incubation of *Pa*‐Cti with palmitoleic acid (16 : 1 *cis*‐Δ^9^) as a substrate. Palmitoleic acid (1 mM) was incubated at 30 °C with *Pa*‐Cti (0.3 μM) in buffer for 18 h. The reaction was quenched using 6 N HCl/MeOH. FAME were analyzed by ^1^H NMR (300 MHz) after 1 h heating at 90 °C followed by extraction with CH_2_Cl_2_. Tests were conducted in the presence of the enzyme (purple), in the presence of enzyme boiled for 5 min at 95 °C (brown), and without enzyme (green). Only the 5.3‐5.4 ppm region corresponding to the vinyl protons of the *cis* and *trans* isomers of 16 : 1 methyl esters is shown.

To test the activity of purified *Pa*‐Cti, an enzymatic assay using ^1^H NMR spectroscopy to analyze the reaction product had to be developed. Therefore, palmitoleic acid (16 : 1 *cis*‐Δ^9^) was incubated with *Pa*‐Cti for 18 h at 30 °C, stopping the reaction by addition of a solution of HCl/MeOH. After further heating of the mixture, FAME were extracted and analyzed by ^1^H NMR (Figure 3).

The comparison of the ^1^H NMR spectrum of the FAME resulting from the reaction in the presence of *Pa*‐Cti with the ^1^H NMR spectra of commercially available 16 : 1 (*cis* and *trans*) FAME reveals a complete conversion of 16 : 1 *cis*‐Δ^9^ into 16 : 1 *trans*‐Δ^9^, confirming that *Pa*‐Cti was active. Indeed, the ^1^H NMR spectra show that the chemical shift of the vinyl proton signals of the *cis* methyl ester is centered at 5.34 ppm, while the chemical shift of the vinyl proton signals of the *trans* methyl ester is centered at 5.38 ppm (Figure 3).

The specific activity of *Pa*‐Cti was further determined by incubating 16 : 1 *cis*‐Δ^9^ and *Pa*‐Cti at 30 °C for 15 min (time chosen to ensure that substrate conversion did not exceed 10 %). Fatty acids were extracted, methylated using (trimethylsilyl)diazo‐methane, and quantified by GC using heptadecanoic acid (17 : 0) as internal standard. They were characterized either by (i) comparing their retention times with those of commercial 16 : 1 (*cis* and *trans*) FAME or (ii) using GC‐MS analysis. *Pa*‐Cti when tested with 1 mM palmitoleic acid, exhibited a specific activity of 0.41±0.03 μmol/min/mg (n=8). This value was close to the maximum specific activity of 0.38 μmol/min/mg observed for the native Cti enzyme of *Pseudomonas* sp. E‐3 reported by Okuyama *et al*.^[13]^ Recently, a full kinetic characterization of Cti from *P. putida* KT2240 was published,^[14]^ where a specific activity of ~0.1 μmol/min/mg was found when reverse micelles of 16 : 1 *cis*‐Δ^9^ were used as substrates. As the protein was extracted from the cytoplasm by sonication, we cannot exclude that the Cti from *P. putida* KT2240 purified by the groups of Park and Chang might still contain some apoenzyme that might be at the origin of the difference in specific activity observed between Cti from *P. putida* KT2240^[14]^ and Cti enzymes from *P. aeruginosa* and *Pseudomonas* sp. E‐3.^[13]^ However, as the bacterial origin of the enzyme differs, it cannot be ruled out that Cti from *P. putida* KT2240 is naturally less active than the enzyme of the pathogenic bacteria *P. aeruginosa*.

To determine whether *Pa*‐Cti is active using other fatty acids than 16 : 1 *cis*‐Δ^9^,18 : 1 *cis*‐Δ^9^ and 18 : 1 *cis*‐Δ^11^ were also tested as substrates. As indicated in Table 1, *Pa*‐Cti exhibited a preference for 18 : 1 *cis*‐Δ^11^, the major fatty acids of the membranes of *P. aeruginosa* that contain 18 : 1 *cis*‐Δ^11^ (~35 %) and 16 : 1 *cis*‐Δ^9^ (~10 %).^[40]^ Furthermore, the proportion of *trans* isomers of 16 : 1 *cis*‐Δ^9^ and 18 : 1 *cis*‐Δ^11^ increased by a factor of 30 and 10, respectively (from 0.10 to 3.05 % and from 0.15 to 1.46 %, respectively, Figure 2) in the experiment intended to detect *in vivo Pa*‐Cti activity, while the proportion of *trans* isomer of 18 : 1 *cis*‐Δ^9^ was not significantly increased (0.03 to 0.04 %) (Figure 2). Therefore, the substrate specificity of purified *Pa*‐Cti is in line with both the analysis of *P. aeruginosa* membrane fatty acid composition and the study of the *in vivo* activity.

**Table 1:** Substrate specificity of *Pa*‐Cti.

Substrate	Relative activity (%)^a^
16 : 1 *cis*‐Δ^9^ (ω‐7)	100
18 : 1 *cis*‐Δ^9^ (ω‐9)	48
18 : 1 *cis*‐Δ^11^ (ω‐7)	146

Enzymatic assays were conducted by incubating *Pa*‐Cti (85 nM) with each respective *cis*‐UFA in buffer (1 mL) for 15 min at 30 °C, and the rate of *trans*‐UFA formation was measured. [a] Activity was determined using different fatty acids as substrates and expressed as a percentage of the activity observed with palmitoleic acid [16 : 1 *cis*‐Δ^9^ (100 %)].

### Phospholipids as Substrates

An unresolved question is whether Cti can utilize phospholipids as substrates as fatty acids are esterified within phospholipids in membranes.^[7]^ According to the literature, no *in vitro* activity was detected when a Cti enzyme was incubated with phospholipids as substrates.[^12^, ^13^, ^41^] Pedrotta *et al*. demonstrated that native Cti from *P. oleovorans* GPo12 exhibited high specificity for free fatty acids.^[12]^ Fatty acid esters were not substrates of the enzyme, even in the presence of organic solvents known to be activators of *cis*‐*trans* isomerization *in vivo*.[^5^, ^42^, ^43^] However, when crude membranes isolated from cells of *P. oleovorans* GPo12 or *E. coli* were used as a source of phospholipids and exposed to the native Cti enzyme, it was reported that the amount of *trans*‐UFAs in the membranes increased, and this increase was significant only in the presence of organic solvents.^[12]^ When phospholipids ([16 : 1 *cis*‐Δ^9^]_2_ – PC and [16 : 1 *cis*‐Δ^9^]_2_ – PE) were tested as substrates by Okuyama *et al*., [16 : 1 *cis*‐Δ^9^]_2_ – PE was isomerized by the native Cti enzyme only in the presence of the membrane fraction prepared from *Pseudomonas* sp. E‐3.[^13^, ^41^]

To investigate whether *Pa*‐Cti could utilize phospholipids as substrates, we developed here an innovative biomimetic model based on liposomes. This approach offers the advantage of a greater control of the assay compared to using crude membranes isolated from cells, which may contain contaminating intrinsic proteins. Hence, we prepared liposomes using two phospholipids, POPC ([16 : 0][18 : 1 *cis*‐Δ^9^] – PC) and POPE ([16 : 0][18 : 1 *cis*‐Δ^9^] – PE), both of which containing oleic acid, following the Bangham method.[^44^, ^45^] Subsequently, we investigated whether *Pa*‐Cti could isomerize phospholipids in this lipophilic environment. Therefore, we conducted an activity test using POPC or POPE, constituents of the liposomes, as substrates. After 18 h incubation at 30 °C, the phospholipids were transesterified using a HCl/MeOH solution and the resulting FAME (*cis* and *trans*) were extracted and analyzed by GC. No isomerization of fatty acids esterified to phospholipids was observed under these conditions (Table 2, Entries 2–4). However, when free 16 : 1 *cis*‐Δ^9^ was added to the assay containing the liposomes, we discovered that *Pa*‐Cti catalyzed the *cis*‐*trans* isomerization of phospholipid's UFAs and of 16 : 1 *cis*‐Δ^9^ (Table 2, Entries 5–7). In line with prior research findings,[^12^, ^13^, ^41^] *Pa*‐Cti does not isomerize phospholipids, both in their free form and incorporated into liposomes (Table 2, Entries 2–4). However, the concurrent presence of free fatty acids and phospholipids not only facilitates the isomerization of free fatty acids, but also allows the isomerization of phospholipid‐bound fatty acids (Table 2, Entries 5–7). These results mark the first direct observation of *in vitro* enzyme activity on phospholipids by a pure Cti enzyme. Nevertheless, the specific role of free fatty acids in this process remains to be clarified.

**Table 2:** Percentage of *trans* fatty acids formed (%) when palmitoleic acid, liposomes, or a mixture of palmitoleic acid and liposomes were used as substrates for *Pa*‐Cti.

Entry	Substrate	Percentage of *trans* fatty acid formed (%)	
		Palmitelaidic acid (16 : 1 *trans*‐Δ^9^)^a^	Elaidic acid (18 : 1 *trans*‐Δ^9^)^b^
**1**	16 : 1 *cis*‐Δ^9^ (1 mM)	80.3±4.5	NA
**2**	POPC (1 mM) without 16 : 1 *cis*‐Δ^9^	NA	0
**3**	POPE (1 mM) without 16 : 1 *cis*‐Δ^9^	NA	0
**4**	POPC (0.5 mM)+POPE (0.5 mM) without 16 : 1 *cis*‐Δ^9^	NA	0
**5**	POPC (0.5 mM) in the presence of 16 : 1 *cis*‐Δ^9^ (0.5 mM)	78.9±3.7	3.3±1.5
**6**	POPE (0.5 mM) in the presence of 16 : 1 *cis*‐Δ^9^ (0.5 mM)	81.3 ±_‐_ 4.6	15.1±3.2
**7**	POPC (0.25 mM)+POPE (0.25 mM) in the presence of 16 : 1 *cis*‐Δ^9^ (0.5 mM)	80.6±5.1	10.8±0.7

Palmitoleic acid (1 mM), liposomes (1 mM), or a mixture of palmitoleic acid (0.5 mM) and liposomes (0.5 mM) in buffer were used to determine the amount of *trans* fatty acids formed after incubation with *Pa*‐Cti (0.3 μM). The reaction mixture (1 mL) was incubated for 18 h at 30 °C. The quantity of *trans* fatty acids formed was determined by GC after transesterification of the phospholipids and/or esterification of the free fatty acids. The values are presented as the mean ± standard deviation of three independent experiments (n=3). POPC: 1‐palmitoyl‐2‐oleoyl‐sn‐glycero‐3‐phosphatidylcholine; POPE: 1‐palmitoyl‐2‐oleoyl‐sn‐glycero‐3‐phosphoethanolamine. [a] Derived from free fatty acid. [b] Derived from phospholipids.

**Figure 4:**
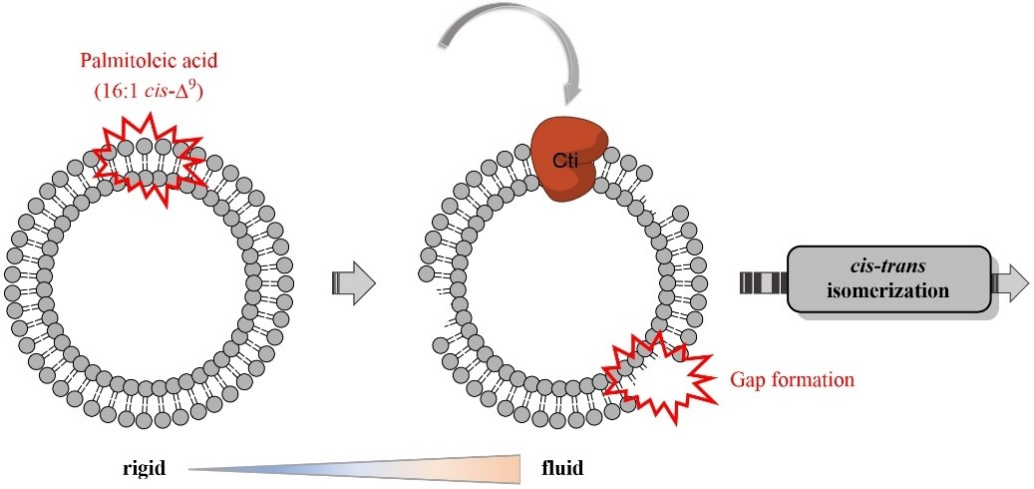
Potential role of free fatty acids in mediating membrane fluidity and facilitating the *cis*‐*trans* isomerization of phospholipid fatty acids in the liposome membrane.

Several studies have shown that free fatty acids can influence the properties of the membrane.[^46^, ^47^, ^48^] Therefore, we hypothesize that the presence of 16 : 1 *cis*‐Δ^9^ in the reaction mixture containing liposomes might alter the arrangement of the lipid chains within the phospholipids. This change in the liposome membrane organization could potentially allow *Pa*‐Cti to access the double bonds of the *cis*‐UFAs within the phospholipids, leading to their conversion into *trans*‐UFAs (Figure 4). These findings align with the proposed regulation of Cti through membrane fluidity, as described in the literature by Heipieper *et al*.^[6]^ According to this model, an increase in fluidity, induced by factors such as hydrocarbons or elevated temperatures, would create gaps in the arrangement of phospholipids, allowing Cti to access *cis*‐UFAs residues and facilitate their conversion into *trans*‐UFAs.

### Raman and IR Spectroscopy

**Figure 5:**
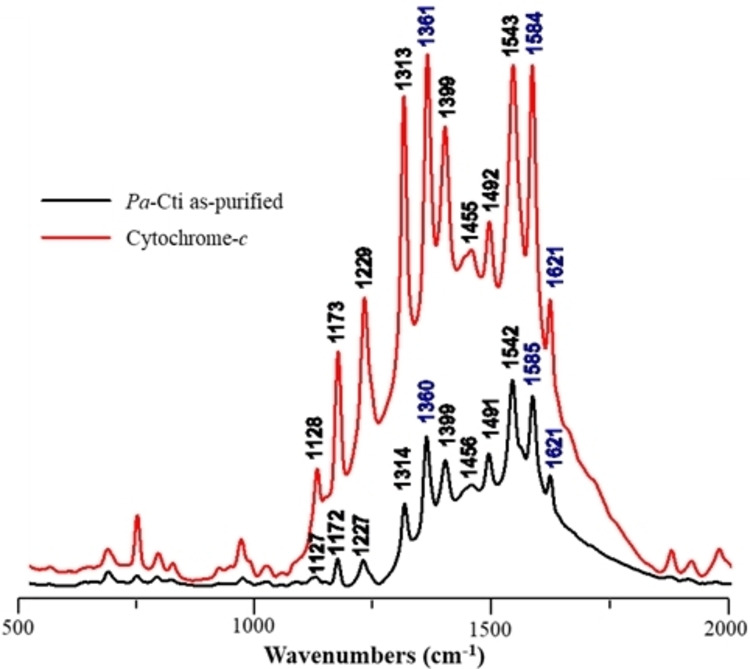
Resonance Raman (RR) spectrum of *Pa*‐Cti. The spectrum was obtained with 514 nm excitation for the purified *Pa*‐Cti (350 μM, black) in K_2_HPO_4_ buffer at pH 8. It was compared to the spectrum of bovine cytochrome‐*c* (1 mM, red) in K_2_HPO4 buffer at pH 7.

The Resonance Raman (RR) spectrum of *Pa*‐Cti, obtained upon excitation at a wavelength of 514 nm, is depicted in Figure 5.

This spectrum has been subjected to a comparative analysis with the RR spectrum of bovine cytochrome‐*c*, revealing a remarkable similarity between both spectra. The observed peaks at 1360 (ν_4_), 1491 (ν_3_), 1585 (ν_2_), and 1621 (ν_10_) cm^−1^ in the spectrum of *Pa*‐Cti are indicative of the heme‐*c* being in a reduced state. There is a substantial body of literature available for the spectroscopic analysis of heme‐containing proteins, such as cytochrome‐*c*,[^49^, ^50^, ^51^, ^52^, ^53^] and comparing the available data with the high‐frequency region bands of the RR spectra of *Pa*‐Cti suggests that the heme iron atom is present as a low‐spin hexacoordinated species (Table 3).

**Table 3:** RR spectroscopy parameters *Pa*‐Cti and horse heart cytochrome‐*c*.

Protein	Wavenumbers (cm^−1^)				Coordination; Spin	Reference
	ν_4_	ν_3_	ν_2_	ν_10_		
*Pa*‐Cti	1360	1491	1585	1621	6; low‐spin	this study
Ferrocytochrome‐*c* (Fe^II^)	1362	1493	1584	1620	6; low‐spin	[49,51]
Ferricytochrome‐*c* (Fe^III^)	1374	1502	1582	1636	6; low‐spin	[49,51]

This RR spectroscopy study provides further evidence for the presence of heme‐Fe^II^ as previously suggested using UV‐Visible spectroscopy and it appears that the iron is hexacoordinated with a low‐spin configuration.

### Redox Potential of Pa‐Cti

**Figure 6:**
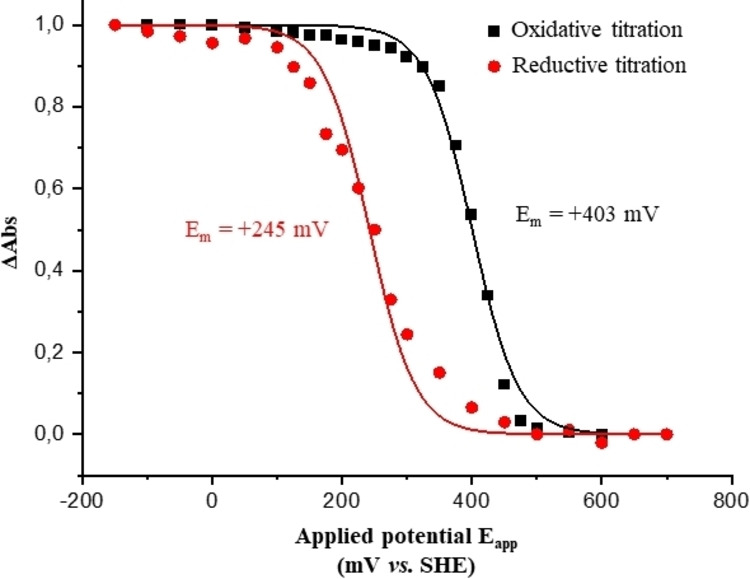
Determination of the mean redox potential (E_m_) of *Pa*‐Cti. The analysis of the *Pa*‐Cti protein (800 μM) in K_2_HPO_4_ buffer pH 8 was carried out in a thin‐layer electrochemical cell. The plot of ▵Abs *vs*. E_app_ was generated using the α band (556 nm). The fitting of the titration curves using the Nernst equation was performed with n=0.5

The redox potential of the heme in *Pa*‐Cti was further determined by coupling UV‐Visible spectroscopy with an electrochemical cell.[^54^, ^55^] An average redox potential (E_m_) of +245 mV and +403 mV were determined for the reductive titration and oxidative titration, respectively (Figure 6).

The results of reductive titration differ from those of the oxidative titration, displaying an unusual hysteresis behavior of 120–150 mV. Also, it is not possible to fit the data using the Nernst equation with n=1, but it fits much better with n=0.5.

Several factors are significantly involved in controlling the redox potential, particularly axial coordination and second‐sphere coordination.[^56^, ^57^] The presence of histidine in the proximal position is a consistent structural motif in type‐*c* cytochromes.[^27^, ^28^, ^29^] Numerous publications have reported the influence of the nature of the sixth axial ligand of different type‐*c* cytochromes on the E_m_ value.[^56^, ^57^, ^58^] Generally, class I cytochromes‐*c*, with His/Met coordination, exhibit E_m_ values ranging from 0 to +400 mV, while class III cytochromes‐*c*, with His/His coordination, have values ranging from −400 to −100 mV.^[56]^ The E_m_ values obtained during potentiometric titration by UV‐Visible spectroscopy for *Pa*‐Cti suggest a heme coordination scheme favoring His/Met coordination.

The unusual hysteresis behavior within the range of 120–150 mV can be attributed to various factors such as cooperativity between the heme‐*c* and other redox centers in the protein. It is also conceivable that this potential shift occurs due to a partial alteration in the heme ligand conformation or other modifications taking place within the protein during the extended duration of the titration process.^[59]^ Another possibility is that the heme‐*c* center within the protein may undergo changes in its coordination environment during the course of titration, akin to what has been observed in other cytochromes‐*c*.[^60^, ^61^] Both the hysteresis and unusual electron stoichiometry (n=0.5) might also be due to slow electron transfer if the heme is not very accessible and deeply buried into the protein. While it is plausible that the hysteresis observed in *Pa*‐Cti could be an outcome of an experimental artifact, it cannot be ruled out at this stage that it might bear biological significance, as demonstrated in the case of the CooA protein from *Rhodospirillum rubrum*.[^62^, ^63^] CooA, a heme‐containing transcriptional activator, exhibits redox anomalies, with hysteresis observed during electrochemical redox titrations where the reduction and oxidation potentials recorded were −320 mV and −260 mV, respectively.[^62^, ^63^] This latter discrepancy in potential between reduction and oxidation is believed to stem from ligand exchange events controlled by the heme's oxidation state between Cys75 and His77, as suggested in the literature.^[63]^ Class I cytochromes‐*c*, with His/Met coordination, have been shown to exist in two different conformations depending on the orientation of the axial methionine, driven by inversion at the methionine sulfur atom.[^64^, ^65^, ^66^] It has been reported that change in redox potential can be associated with this conformational alteration of the axial methionine side chain.^[67]^ At this stage, it cannot be excluded that heme axial methionine fluxionality occurs in *Pa*‐Cti during the titration process, leading to hysteresis.

### Mössbauer Spectroscopy Analysis of the Heme of *Pa*‐Cti

**Figure 7:**
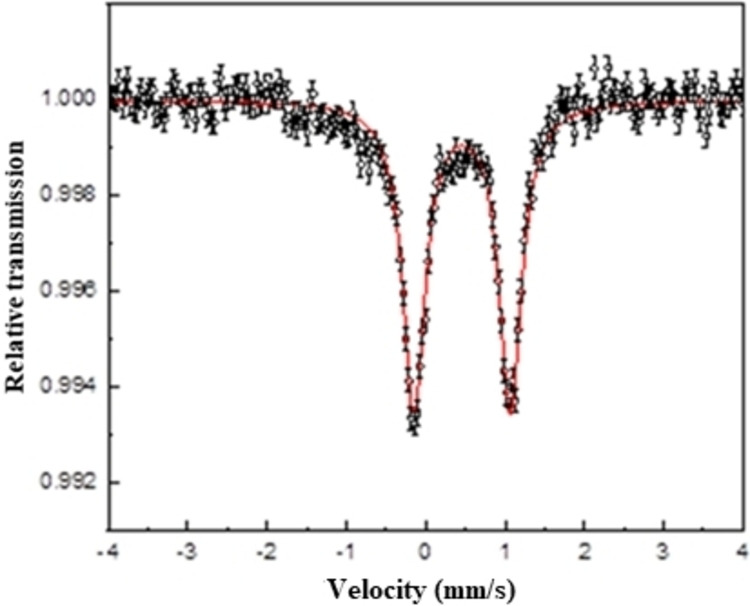
Mössbauer spectrum of ^57^Fe‐labeled *Pa*‐Cti recorded at 77 K. *Pa*‐Cti concentration was 650 μM. The red solid line is the result of a fitting procedure leading to δ =0.46(1) mm/s, ΔE_Q_=1.22(1) mm/s, and Γ=0.31(1) mm/s.

To ascertain that *Pa*‐Cti was isolated in the low spin ferrous state and to have more hints on the coordination sphere of this iron, Mössbauer spectroscopy investigations were carried out on the ^57^Fe‐labeled *Pa*‐Cti (Figure 7).

The Mössbauer spectrum measured at 77 K exhibits a doublet with an isomer shift (δ ) of 0.46 mm/s and a quadrupole splitting (ΔE_Q_) of 1.22 mm/s. These Mössbauer parameters when compared to those reported for other heme containing proteins[^68^, ^69^, ^70^, ^71^] (Table 4) highlight that *Pa*‐Cti resembles the most to cytochrome‐*c*_552_.^[71]^ It can be noticed that the Mössbauer parameters for *Pa*‐Cti differ drastically from those of a pentacoordinated high‐spin Fe^II^ heme, such as typically found in deoxymyoglobin.^[69]^ This Mössbauer spectroscopy study firmly confirms the presence in *Pa*‐Cti of an octahedral low‐spin ferrous iron (Fe^II^, S=0).

**Table 4:** Mössbauer spectroscopy parameters for *Pa*‐Cti and various heme‐*c*.

Protein	Isomer shift δ (mm/s)	Quadrupole splitting ΔE_Q_ (mm/s)	Coordination; Spin	Reference
Cti as‐purified	0.46	1.22	6; Fe^II^ low‐spin	This study
Cytochrome‐*b_6_*	0.20	1.77	6; Fe^III^ low‐spin	[70]
Cytochrome‐*f*	0.26	1.90	6; Fe^III^ low‐spin	[70]
Cytochrome‐*c*_552_	0.46	1.30	6; Fe^II^ low‐spin	[71]
Deoxymyoglobin	0.89	2.19	5; Fe^II^ high‐spin	[69]

In cytochrome‐*c*_552_, iron is axially coordinated with a histidine from the CXXCH heme‐binding motif and a methionine as the sixth ligand.^[71]^ Therefore, based on the similarity in Mössbauer parameters between *Pa*‐Cti and cytochrome‐*c*_552_, along with the measured E_m_ values, it is reasonable to propose that a methionine serves as the sixth distal ligand of the heme iron in *Pa*‐Cti, alongside the proximal ligand histidine

### Met163 of Pa‐Cti is the Distal Ligand of the Heme

Multiple sequence alignments using the amino acid sequences of several Cti proteins (Figure S5 and Table S1) revealed a single conserved methionine corresponding to Met163 in the recombinant *Pa*‐Cti sequence. We hypothesized that Met163 could potentially act as the distal axial ligand. Therefore, mutants of *Pa*‐Cti were generated by replacing the corresponding methionine by an alanine or a histidine residue using site‐directed mutagenesis, and the corresponding *Pa*‐Cti mutants were produced and purified as described for *Pa*‐Cti wild‐type (WT).

The activity of *Pa*‐Cti M163A and *Pa*‐Cti M163H mutants were further assessed using 16 : 1 *cis*‐Δ^9^ as substrate, and ^1^H NMR and GC analyses for detection of the FAME according to the methodologies previously established for *Pa*‐Cti WT (Figure S6 and Table 5). The ^1^H NMR spectrum of the FAME arising from the assay of *Pa*‐Cti M163H did not reveal any characteristic signals

**Table 5:** Percentage of *trans*‐fatty acid formed (%) after incubation of *Pa*‐Cti WT, *Pa*‐Cti M163A or *Pa*‐Cti M163H with palmitoleic acid as substrate.

Protein	Percentage of *trans* fatty acid formed (%)
*Pa*‐Cti WT	80
*Pa*‐Cti M163A	26
*Pa*‐Cti M163H	1

Assays were performed by incubating *Pa*‐Cti WT (0.3 μM), *Pa*‐Cti M163A (0.3 μM), or *Pa*‐Cti M163H (0.3 μM) with palmitoleic acid (1 mM) in buffer. The reaction mixture (1 mL) was incubated for 18 h at 30 °C. The amount of *trans*‐fatty acid formed was determined by GC after derivatization of the fatty acids into methyl esters. The percentage was calculated by dividing the amount of *trans* fatty acid formed by the amount of total 16 : 1 fatty acids present in the assay.

of the *trans* isomer. The inactivity of this mutant was further confirmed using GC analysis. Conversely, the *Pa*‐Cti M163A mutant appeared to retain some activity as a signal characteristic of the *trans* isomer of 16 : 1 *cis*‐Δ^9^ with δ=5.38 ppm was present on the ^1^H NMR spectrum. Further quantification of FAME by GC indicated that this mutant converts around 26 % of the substrate after 18 h of incubation. These results revealed that the mutation of Met163 has an impact on the activity of *Pa*‐Cti. The fact that the *Pa*‐Cti M163A mutant retains some activity suggests that the mutation did not render the enzyme completely non‐functional.

**Figure 8:**
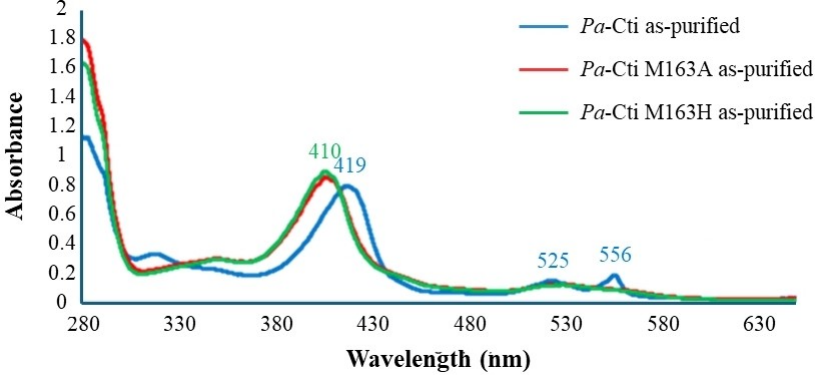
UV‐Visible absorption spectra of WT *Pa‐*Cti, *Pa*‐Cti M163A and *Pa*‐Cti M163H. 12 μM (~1 mg mL^−1^) solution of purified *Pa*‐Cti (blue), *Pa*‐Cti M163A (red) and *Pa*‐Cti M163H (green).

Contrary to the UV‐Visible absorption spectrum of *Pa*‐Cti WT revealing a reduced type‐*c* cytochrome, both *Pa*‐Cti M163A and *Pa*‐Cti M163H exhibit a heme iron in the ferric state, with a shift in the Soret absorption band from 419 to 410 nm (Figure 8). Due to this difference in oxidation state between the wild‐type and the mutants, spectroscopic UV‐Visible titrations coupled with electrochemistry[^54^, ^55^] were performed for *Pa*‐Cti M163A and *Pa*‐Cti M163H using the methodology previously established for *Pa*‐Cti WT. The titration curves are displayed in Figure S7. Interestingly, for both mutants curve fitting could be performed based on the Nernst equation with here n=1 and no hysteresis was observed for neither of the mutants.

Electrochemical characterization of *Pa*‐Cti M163H revealed an E_m_ value of −181 mV (Table 6), in accordance with His/His coordination. Investigation of *Pa*‐Cti M163A yielded results akin to those of *Pa*‐Cti M163H, albeit with a more pronounced reduction in the E_m_ value (approximately −450 mV to −650 mV in comparison to the wild‐type, Table 6). The M163A mutation was purposefully introduced to establish a coordination site that could potentially (i) remain vacant, (ii) be occupied by water (or hydroxide), or (iii) be substituted by another amino acid of *Pa*‐Cti. For structurally characterized type‐*c* cytochromes, heightened solvent exposure significantly diminishes the observed heme redox potential, with a decrease estimated to be around −240 mV in comparison to its presence in a hydrophobic environment as reported by Tezcan *et al*.^[72]^ In the case of *Pa*‐Cti M163A, the decrease exceeds −400 mV so other structural elements may also contribute. However, it is important to consider the possibility that other amino acids might also have the potential to replace the thioether bond of Met163 in *Pa*‐Cti M163A, as has been observed in eukaryotic cytochrome‐*c* proteins.[^73^, ^74^] This potential substitution could account for the diminished activity of Cti M163A.

**Table 6:** Average redox potential of WT *Pa*‐Cti, *Pa*‐Cti M163A and *Pa*‐Cti M163H.

Protein	Average redox potential E_m_ [mV (*vs*. SHE)]	
	Oxidative titration	Reductive titration
*Pa*‐Cti WT	+403	+245
*Pa*‐Cti M163A	−247	−216
*Pa*‐Cti M163H	−182	−181

Taken together, our investigations revealed that Met163 serves as the sixth ligand of the iron in *P. aeruginosa* Cti. Importantly, the *Pa*‐Cti M163A mutant exhibited a diminished activity, suggesting that the catalytic mechanism of Cti may operate independently of the oxidation state of the heme iron.

## Conclusions

This study represents a significant advancement in our understanding of the Cti metalloenzyme from *P. aeruginosa*. The successful optimization of protein production, purification, and characterization has provided a platform for future research into this intriguing enzyme. The development of innovative enzymatic assays and the demonstration of Cti activity on phospholipids within a biomimetic context mark an important milestone. Our spectroscopic analyses, including resonance Raman and Mössbauer spectroscopy, have shed light on the heme coordination and redox properties of Cti. Identification of Met163 as the sixth ligand of the heme iron in *P. aeruginosa* Cti furthers our knowledge of its structure and function. Moreover, the unexpected finding of some activity for *Pa*‐Cti M163A raises intriguing questions about the enzyme catalytic mechanism, hinting at potential complexity beyond the iron oxidation state. These findings not only expand our comprehension of Cti but also lay the foundation for future studies that may include crystallography and additional biophysical techniques. Ultimately, this work paves the way for a more comprehensive understanding of the role of Cti in bacterial lipid metabolism and its potential implications in antimicrobial resistance.

## Conflict of Interests

The authors declare no conflict of interest.

1

## Supporting information

As a service to our authors and readers, this journal provides supporting information supplied by the authors. Such materials are peer reviewed and may be re‐organized for online delivery, but are not copy‐edited or typeset. Technical support issues arising from supporting information (other than missing files) should be addressed to the authors.

Supporting Information

## Data Availability

The data that support the findings of this study are available from the corresponding author upon reasonable request.
