# Supplementary material for: Towards Bacterial Resistance via the Membrane Strategy: Enzymatic, Biophysical and Biomimetic Studies of the Lipid cis‐trans Isomerase of Pseudomonas aeruginosa
Source: Chembiochem. 2024 Nov 28;26(1):e202400844. doi: 10.1002/cbic.202400844 (PMC11727003; doi:10.1002/cbic.202400844)
Supplement: Supplementary file 1 — Supporting Information [file CBIC-26-e202400844-s001.pdf]

ChemBioChem

Supporting Information

Towards Bacterial Resistance *via* the Membrane Strategy: Enzymatic, Biophysical and Biomimetic Studies of the Lipid *cis-trans* Isomerase of *Pseudomonas aeruginosa*

Mickaël Mauger, Iryna Makarchuk, Yasmin Molter, Anna Sansone, Frédéric Melin, Philippe Chaignon, Philippe Schaeffer, Pierre Adam, Volker Schünemann, Petra Hellwig, Carla Ferreri, Chryssostomos Chatgilialoglu, and Myriam Seemann*

Towards Bacterial Resistance *via* the Membrane Strategy: Enzymatic, Biophysical and Biomimetic Studies of the Lipid *cis-trans* Isomerase of *Pseudomonas aeruginosa*

Mickaël Mauger,[a] Iryna Makarchuk,[b] Yasmin Molter,[c] Anna Sansone,[d] Frédéric Melin,[b] Philippe Chaignon,[a] Philippe Schaeffer,[e] Pierre Adam,[e] Volker Schünemann,[c] Petra Hellwig, [b,f] Carla Ferreri,[d] Chryssostomos Chatgilialoglu,[d,g] Myriam Seemann*[a]

[a] Dr. M. Mauger, Dr. P. Chaignon, Dr. M. Seemann
Equipe Chimie Biologique et Applications Thérapeutiques, Institut de Chimie de Strasbourg UMR 7177, Université de Strasbourg, CNRS, 67000 Strasbourg, France
E-mail: mseemann@unistra.fr

[b] Dr. I. Makarchuk, Dr. F. Melin, Prof. P. Hellwig
Laboratoire de Bioélectrochimie et Spectroscopie, Chimie de la Matière Complexe UMR 7140, Université de Strasbourg, CNRS, 67000 Strasbourg, France

[c] Y. Molter, Prof. V. Schünemann
Department of Physics, University of Kaiserslautern-Landau, Erwin-Schrödinger-Str. 46, 67663 Kaiserslautern, Germany

[d] Dr. A. Sansone, Dr. C. Ferreri, Dr; C. Chatgilialoglu
Institute for Organic Synthesis and Photoreactivity, National Research Council, 40129 Bologna, Italy

[e] Dr. P. Schaeffer, Dr. P. Adam
Equipe Biogéochimie Moléculaire, Institut de Chimie de Strasbourg UMR 7177, Université de Strasbourg, CNRS, 67000 Strasbourg, France

[f] Prof. P. Hellwig
Institut Universitaire de France (IUF)

[g] Dr. C. Chatgilialoglu
Center for Advanced Technologies, Adam Mickiewicz University, 61-614 Poznań, Poland

Table of Contents

Experimental section 2
Figure S1: Analysis of *Pa*-Cti production expressed in *Escherichia coli* BL21(DE3) using the pET22b vector containing the *cti* gene and in the presence of the pEC86 vector containing the *cytochrome*-c *maturation genes* 5
Figure S2: 10% SDS-PAGE analysis of *Pseudomonas aeruginosa* Cti production in the presence of cytochrome-*c* maturation genes 6
Figure S3: Western blot analysis of basal *Pa*-Cti expression after induction of the *cis-trans* isomerization reaction of UFA by octanol 7
Figure S4: Distribution of membrane fatty acids in *Escherichia coli* BL21(DE3)[pET22b/*Pa-cti*; pEC86] cultured at 30 °C 7
Figure S5: Multiple sequence alignment of the Cti proteins 8
Table S1: BLAST analysis based on the Cti protein sequence of *Pseudomonas aeruginosa* PAO1 8
Figure S6: ^{1}H NMR spectra (300 MHz) of FAMEs obtained after incubation of *Pa*-Cti WT or the mutants *Pa*-Cti M163A and *Pa*-Cti M163H with palmitoleic acid (16:1 *cis*-Δ^9) as substrate 9
Figure S7: Determination of the average redox potential (E_m) for the mutants *Pa*-Cti M163A and *Pa*-Cti M163H 9
Figure S8: Differential infrared spectra of *Pa*-Cti 10
Table S2: Tentative assignment of the observed bands in the differential infrared spectrum of the reduced form minus the oxidized form of *Pa-Cti* 10
Table S3: Plasmids 11
Table S4: Oligonucleotides 11
Figure S9: (a.) Coding sequence and (b.) deduced protein sequence of Cti from *Pseudomonas aeruginosa* in the pET30b expression vector 13
Figure S10: (a.) Coding sequence and (b.) deduced protein sequence of Cti from *Pseudomonas aeruginosa* in the pET22b expression vector 13
References 13

Experimental section

Materials

Oleic acid (≥ 99%), palmitoleic acid (≥ 98.5%), *cis*-vaccenic acid (≥ 97%), elaidic acid (> 99%), *trans*-vaccenic acid (> 99%) were purchased from Sigma-Aldrich Co. Methyl oleate (≥ 99%), methyl elaidate (≥ 99%), *cis*-11-octadecenoic methyl ester (analytical standard grade), methyl *trans*-vaccenate (≥ 99%) were purchased from Sigma-Aldrich Co. Palmitelaidic acid, methyl palmitoleate and palmitelaidic acid methyl ester were purchased from Cayman Chemical Co. Heptadecanoic acid (≥ 98%) and docosanoic acid (analytical standard grade) were purchased from Sigma-Aldrich Co. 1-Palmitoyl-2-oleoyl-sn-glycero-3-phosphocholine (POPC) and 1-palmitoyl-2-oleoyl-sn-glycero-3-phosphoethanolamine (POPE) were purchased from Avanti Polar Lipids. Ampicillin sodium salt, kanamycin sulfate, isopropyl β-D-1-thiogalactopyranoside (IPTG) were purchased from Euromedex. Chloramphenicol (≥ 98%), iron chloride (anhydrous powder, ≥ 99.9% trace metals basis), 3,3',5,5'-tetramethylbenzidine (≥ 99%) were purchased from Sigma-Aldrich Co. High performance liquid chromatography-grade n-hexane (ReagentPlus, ≥ 99%), methanol (≥ 99%), and other chemicals (extra pure grade) were purchased from Sigma-Aldrich Co.

Bacterial strains, plasmids and oligonucleotides

The plasmids and oligonucleotides used in this work are listed in **Table S3** and **Table S4**, respectively. All oligonucleotides were synthesized and supplied by Sigma-Aldrich Co. The concentration of stock solutions was 100 µM. The *E. coli* XL1-Blue and BL21(DE3) strains were employed as hosts for cloning and overexpression, respectively. If needed, PCR products and plasmids were purified using the QIAquick Gel Extraction Kit (Qiagen) and QIAprep Spin Miniprep Kit (Qiagen), respectively. Restriction enzymes were purchased and utilized according to the manufacturer instructions (Promega). Ligation was performed with T4 DNA ligase following the manufacturer recommendations (Invitrogen).

Cloning of *Pseudomonas aeruginosa cti* gene into the expression vector pET22b

The pET30b/*Pa*-cti vector encoding the entire *Pa*-Cti (with the native peptide signal) after codon optimization was provided by GenScript **(Fig. S9)**. Using this plasmid as a template, recognition sequences for the NcoI and XhoI restriction enzymes were respectively introduced at the 5'- and 3'- ends of the sequence during PCR to generate a gene fragment encoding *P. aeruginosa* Cti without the native signal peptide. After digestion of the fragment and of pET22b plasmid with NcoI and XhoI, the fragment was inserted into the respective sites of pET22b expression vector. The reaction mixture treated with T4 DNA ligase was incubated (18 h, 16 °C), and a fraction of this mixture (5 µL) was introduced by heat shock into competent *E. coli* XL1-Blue cells. The plasmid DNA pET22b/*Pa-cti* amplified by *E. coli* XL1-Blue[pET22b/*Pa-cti*] was extracted and purified using the QIAprep Spin Miniprep Kit (Qiagen). The pET22b/*Pa-cti* plasmid was recovered (50 µL, ~250 ng/µL, ~0.1 pmol/µL) and analyzed by 0.5% agarose gel electrophoresis to verify its integrity. The accuracy of the DNA sequence of the *cti* gene was confirmed through sequencing **(Fig. S10)**. The pET22b/*Pa-cti* plasmid was introduced by heat shock into competent *E. coli* BL21(DE3) (or *E. coli* BL21(DE3)[pEC86]) cells, generating expression strains *E. coli* BL21(DE3)[pET22b/*Pa-cti*] (or BL21(DE3)[pET22b/*Pa-cti*; pEC86]).

Site-Directed Mutagenesis

The plasmid pET22b/*Pa-cti*, encoding the recombinant Cti protein from *P. aeruginosa* with a pelB *N*-terminal signal peptide and a *C*-terminal His_6-tag, serves as the DNA template **(Fig. S10)**. The primers listed in **Table S4** were employed to introduce the desired mutation using the QuikChange Lightning Site-Directed Mutagenesis Kit (Agilent) and following the manufacturer instructions. The plasmid DNA pET22b/*Pa-cti* M/A(H) amplified by *E. coli* XL10-Gold[pET22b/*Pa-cti* M/A(H)] was extracted and purified using the QIAprep Spin Miniprep kit (Qiagen). The pET22b/*Pa-cti* M/A(H) plasmids were recovered (50 µL, ~250 ng/µL, ~0.1 pmol/µL) and analyzed by 0.5% agarose gel electrophoresis. The presence of the desired mutation and the overall accuracy of the sequence were confirmed through sequencing. The pET22b/*Pa-cti* M/A(H) plasmids were introduced into competent *E. coli* BL21(DE3)[pEC86] cells through a heat shock procedure, resulting in expression strains *E. coli* BL21(DE3)[pET22b/*Pa-cti* M/A(H); pEC86].

SDS-PAGE, Western blot and peroxidase activity detection

For purity analysis, protein samples were mixed with Laemmli loading buffer and heated (5 min, 95 °C). An aliquot of the samples (10 µL, ~0.4 mg/mL) was then loaded onto a 10% SDS-PAGE gel. After migration, the gel was stained with Coomassie Blue (GelCode Blue Safe Protein Stain 24596, Thermo Fisher Scientific).

If a Western blot was planned, a colored molecular weight marker [Precision Plus Protein WesternC Standards (Bio-Rad) or PageRule Plus Pre-stained Protein Ladder (Thermo Fisher Scientific)] was loaded on the 10% SDS-PAGE gel as a transfer control. The transfer was performed onto a polyvinylidene difluoride (PVDF) membrane (Trans-Blot Turbo Mini 0.2 µm PVDF Transfer Packs, Bio-Rad) using the Trans-Blot Turbo Transfer System (Bio-Rad). The membrane was then incubated with gentle shaking (1 h, 25 °C) in TBST saturation buffer [20 mL, 20 mM Tris-HCl, 137 mM NaCl, 0.1% (v/v) Tween 20 pH 7.5] containing skimmed milk (5%). After removing the saturation buffer, the membrane was incubated with gentle shaking (1 h, 25 °C) in TBST saturation buffer (10 mL) containing skimmed milk (5%)

and the primary antibody [5 µL of 6X His tag antibody (GT359) 1.76 mg/mL, Genetex]. After washing the membrane with TBST buffer (3 x 25 mL), TBST saturation buffer (10 mL) with skimmed milk (5%) and the secondary antibody [60 µL of Goat Anti-Mouse IgG antibody (HRP) 0.2 mg/mL, Genetex] were added, and the membrane was further incubated with gentle shaking (1 h, 25 °C). Washing steps were performed with TBST buffer (3 x 25 mL) and distilled water (2 x 25 mL) before detection using the substrate (Novex HRP Chromogenic Substrate, Invitrogen).

For peroxidase activity detection, a colored molecular weight marker [Precision Plus Protein WesternC Standards (Bio-Rad), or PageRule Plus Pre-stained Protein Ladder (Thermo Fisher Scientific)], and horse heart cytochrome-*c* were loaded on the 10% SDS-PAGE gel as controls. Protein samples were mixed with loading buffer [60 mM Tris-HCl pH 6.8, 2% (w/v) SDS, 10% (v/v) glycerol, 0.01% (w/v) bromophenol blue], and an aliquot of the samples (10 µL, ~0.4 mg/mL) was analyzed by SDS-PAGE. A stock solution of 3,3',5,5'-tetramethylbenzidine [TBMZ (6.3 mM)] was prepared in methanol immediately before staining, and 3 volumes (6 mL) of the TBMZ stock solution were mixed with 7 volumes (14 mL) of a sodium acetate solution (0.25 M AcONa pH 5) to produce the TBMZ staining solution. After migration, the gel was incubated with gentle shaking (30 min, 25 °C) in TBMZ staining solution (20 mL), then a 30% H_2O_2 solution (60 µL) was added and the gel was further incubated with gentle shaking for an additional 30 min.

Expression and purification of *Pa*-Cti (or mutants) from *E. coli* BL21(DE3)[pET22b/*Pa-cti*; pEC86] (or *E. coli* BL21(DE3)[pET22b/*Pa-cti* M/A(H); pEC86]

A freshly transformed colony was inoculated into LB medium (5 mL) supplemented with ampicillin (100 µg/mL) and chloramphenicol (25 µg/mL). The culture was incubated with shaking (8 h, 37 °C, 200 rpm) before being transferred to preheated LB culture medium (1 L, 25 °C) supplemented with ampicillin (100 µg/mL) and chloramphenicol (25 µg/mL). The culture was incubated with gentle shaking (25 °C, 125 rpm) until OD_{600} reaches 0.9. The medium was then supplemented with $FeCl_3$ (80 µM) [or $^{57}FeCl_2$ (80 µM) 2 h before induction] and protein expression was induced by adding IPTG (100 µM). The culture was further incubated with gentle shaking (4 – 5 h, 25 °C, 125 rpm) before cells were harvested by centrifugation (20 min, 4 °C, 4000 *x* g). The cell pellet was washed with cold buffer (350 mL, 20 mM Tris-HCl pH 8). All subsequent steps were performed at 4 °C. After centrifugation (20 min, 4 °C, 4000 *x* g), cells (3.5 g) were resuspended in a cold buffer containing sucrose [30 mL, 200 mM Tris-HCl, 1 mM EDTA, 20% (w/v) sucrose pH 8, volume corresponding to 3% of the initial culture volume (1 L)] and lysozyme (1 mL of 30 mg/mL solution), followed by cold ultrapure water [30 mL, corresponding to 3% of the initial culture volume (1 L)] were added. The resulting cell suspension containing 100 mM Tris-HCl, 10% (w/v) sucrose, 0.5 mM EDTA, 0.5 mg mL^{-1} lysozyme at pH 8 was statically incubated (30 min, 25 °C). The generated spheroplasts were separated by centrifugation (25 min, 4 °C, 15000 *x* g), and the supernatant was concentrated on a 50 kDa cutoff filter, filtered through a 0.22 µm syringe filter, and dialyzed overnight with lysis buffer (50 mM Na_2HPO_4, 300 mM NaCl pH 8). The supernatant (~30 – 40 mL) was loaded onto a Ni^{II}-NTA column (Histrap-FF 1 mL, Cytiva) equilibrated with lysis buffer. The resin was first washed (flow rate: 1 mL/min) with a 20 mM imidazole in lysis buffer, and then with a discontinuous imidazole gradient (20 then 150 mM) in lysis buffer. After SDS-PAGE electrophoresis, fractions containing the *Pa*-Cti protein with estimated purity greater than 90% were pooled and concentrated on a 50 kDa cutoff filter. The protein solution was further loaded onto a desalting column (PD-10, Cytiva) eluted with lysis buffer and the recovered protein solution was frozen in liquid nitrogen and stored at -80 °C until further use. The protein concentration was determined using the Bradford method.[1] The apparent molecular weight was determined by gel filtration chromatography on a Hiload 16/200 Superdex 200 Prep-Grade Column (Cytiva) eluted with 50 mM Tris-HCl, 50 mM NaCl pH 8.

***In vivo Pa*-Cti activity**

After transformation of *E. coli* BL21(DE3)[pEC86] cells with the pET22b/*Pa-cti* plasmid (or pET22b), clones were grown in LB medium (1 mL) supplemented with ampicillin (100 µg/mL) and chloramphenicol (25 µg/mL) for 1 h at 37 °C with shaking (200 rpm). A fraction of the culture (50 µL) was spread on a Petri dish containing LB agar supplemented with ampicillin (100 µg/mL), chloramphenicol (25 µg/mL), and IPTG (10 µM) and the plate was further incubated 24 h at 37 °C. A colony was then transferred to LB medium (5 mL) supplemented with ampicillin (100 µg/mL), chloramphenicol (25 µg/mL), and IPTG (10 µM) and the preculture was incubated at 37 °C for 1 h with shaking (200 rpm). A portion of this preculture (1 mL) was added to LB medium (100 mL) supplemented with ampicillin (100 µg/mL), chloramphenicol (25 µg/mL) and IPTG (10 µM). The culture was incubated at 30 °C with shaking (200 rpm). When the OD_{600} reached 0.6, cellular stress was induced by adding octanol [40 µL, 0.04% (v/v) or 2.5 mM]. After incubation (2 h) at 30 °C with shaking (200 rpm), trichloroacetic acid [TCA (10 mL)] was added to immediately stop bacterial metabolism. Cells were harvested by centrifugation (20 min, 4 °C, 4000 *x* g) and the resulting cell pellet was washed with ultrapure water (3 x 50 mL), weighed, frozen, lyophilized. The pellet was treated with a 0.5 N KOH/MeOH solution (0.5 mL) for 1 h at room temperature to obtain fatty acid methyl esters that were isolated and analyzed as previously described.[2]

***In vitro Pa*-Cti activity (with detection of the product by ^1H NMR spectroscopy)**

The enzymatic reaction was initiated by adding the enzyme (0.3 µM) to palmitoleic acid [16:1 *cis*-Δ^9 (1 mM)] in 50 mM Na_2HPO_4, 300 mM NaCl buffer at pH 8 (4 mL). After incubation (18 h, 30 °C), the reaction was stopped by adding a 6 N HCl/MeOH solution (4 mL). The mixture was then heated (120 min, 90 °C), and methyl esters were extracted with CH_2Cl_2 (3 x 8 mL). The organic phases were further combined, dried over anhydrous Na_2SO_4, filtered, and concentrated under vacuum. The crude residue was dissolved in

deuterated chloroform ($CDCl_3$) and analyzed by ^1H NMR using a Bruker AC 300 MHz instrument. Chemical shifts (δ) are reported in parts per million (ppm) relative to chloroform (7.26 ppm) as the internal reference. The NMR spectra resulting from reactions in the presence of *Pa*-Cti enzyme or mutants were compared to reference spectra of commercially available methyl esters of palmitoleic acid and palmitelaidic acid.

In vitro Pa-Cti activity (with detection of the product by GC)

The enzymatic assay was initiated by adding the enzyme to palmitoleic acid [16:1 *cis*-Δ^9 (1 mM)] in 50 mM Na_2HPO_4, 300 mM NaCl buffer at pH 8 (1 mL). After incubation at 30 °C, a 30% (w/v) trichloroacetic acid solution in water (100 µL) was added to quench the reaction followed by the addition of the internal standard (heptadecanoic acid). Free fatty acids were extracted with hexane (3 x 1 mL), the organic layers were combined, dried using 2,2-dimethoxypropane (100 µL) as a water scavenger,[3] and the solvent was removed under a nitrogen flow at 40 °C. For derivatization of the fatty acids prior GC analysis, the residue was dissolved in a toluene/methanol solution [1:2 (1 mL)], to which an excess solution of 2 M TMS-DM in hexane (100 µL) was added. After incubating the reaction at 40 °C for 30 min, excess solvent and TMS-DM were removed under a gentle nitrogen flow at 40 °C. Finally, the residue was dissolved in hexane (1 mL), and a portion of the solution (1 µL) was analyzed by GC using a HP GC 6890 Series gas Chromatograph (Agilent) equipped with a flame ionization detector maintained at 310 °C and an on-column injector used in the 'track-oven' mode. A HP-5 capillary column (30 m x 0.32 mm x 0.25 µm; Agilent) was employed with hydrogen as the carrier gas at a constant flow rate of 2.5 mL/min. Heating of the oven was carried out as follows: 40 °C – 240 °C (4 °C min^{-1}), 240 °C – 300 °C (10 °C min^{-1}), and isothermal at 300 °C for 20 min.

In vitro Pa-Cti activity on phospholipids

A solution of POPC (76 mg, 0.1 mmol), POPE (72 mg, 0.1 mmol), or a mixture of POPC (38 mg, 0.05 mmol) and POPE (36 mg, 0.05 mmol) in $CHCl_3$ (1 mL) was evaporated under an argon flow and then under vacuum for 30 min to form a thin film. Phosphate buffer (1 mL, 50 mM Na_2HPO_4, 300 mM NaCl pH 8) was added, and MLVs were formed by shaking 10 min using a Vortex. To obtain LUVs, the lipid emulsion POPC (100 mM), POPE (100 mM), or POPC/POPE [1:1 (100 mM)] was transferred to a Mini Extruder (Avanti Polar Lipids) and extruded 20 times back and forth through two polycarbonate membranes with 100 nm pores. Palmitoleic acid [16:1 *cis*-Δ^9 (1 mM)], liposomes (1 mM), or a mixture of palmitoleic acid [16:1 *cis*-Δ^9 (0.5 mM)] and liposomes (0.5 mM) were then used to determine the amount of *trans*-fatty acid formed by incubating the *Pa*-Cti enzyme (0.3 µM) in phosphate buffer at pH 8. The reaction mixture (1 mL) was incubated (18 h, 30 °C), and the reaction was stopped by adding a 6 N HCl/MeOH solution (1 mL). The mixture was further heated (120 min, 90 °C), and methyl esters were extracted with CH_2Cl_2 (3 x 8 mL). The organic phases were further combined, dried over anhydrous Na_2SO_4, filtered, and concentrated under vacuum. The crude residue was solubilized in hexane (1 mL), and a portion of the solution (1 µL) was analyzed by GC as previously described.[2]

Mössbauer spectroscopy

Solution of $^{57}FeCl_2$ was prepared as described for $^{57}FeCl_3$,[4] but heating of the reaction was performed under argon atmosphere. A solution of ^{57}Fe-*Pa*-Cti (650 µM, 150 µL) in 50 mM Na_2HPO_4, 300 mM NaCl pH 8 was transferred to a Mössbauer sample holder and frozen in liquid nitrogen until measurement. The Mössbauer spectrum was recorded in the constant acceleration mode with a conventional spectrometer and a multi-channel analyzer in the time-scale mode (WissEL GmbH). Mössbauer spectra were taken at 77 K using a bath cryostat cooled with liquid nitrogen (Oxford Instruments). After data transfer from the multi-channel analyzer to a PC, the public domain program Vinda[5] running on an Excel 2003 platform was used for data analysis. The Mössbauer spectra were analyzed by least-squared fits using Lorentzian line shapes with the line width at half maximum Γ. Isomer shifts δ are given relative to α-iron at room temperature.

Resonance Raman spectroscopy

The Raman scattering spectra were recorded using a Renishaw InVia Reflex Raman spectrometer at 514 nm laser excitation (12.5 mW) with a focus by a Leica 20×/0.40 NA dipping microscope objective. The RR spectra of the protein dry films on CaF_2 windows were obtained from 10 µL of the heme proteins solution (bovine cytochrome-*c*, *Pa*-Cti,) in the corresponding buffer. The collection of the Raman scattering emission was conducted at x50 magnification under 180 ° (backscattering), spectral resolution is 1 cm^{-1}. To obtain spectra of the proteins in the range from 100 to 3200 cm^{-1} with an optimal signal to noise ratio, 20 spectrum accumulations from 3 different spots were made followed by baseline correction.

Redox induced infrared spectroscopy

The differential redox IR spectra of *Pa*-Cti were recorded in a thin-layer electrochemical cell[6] inserted in Vertex 70 Spectrometer (Brucker Optiks) connected to the potentiostat and the cooling system set at 10 °C. In this 3-electrode cell, a 6 µm-thick 70% optically transparent gold grid modified with a 1:1 solution of cysteamine and mercaptopropionic acid was used as working electrode, a platinum wire as a counter electrode and an Ag/AgCl as a reference electrode. The protein solution (5 µL) was deposited on the gold grid and

the cell was closed with CaF_2 windows, then filled with K_2HPO_4 pH 8 buffer containing 100 mM KCl. The redox reaction was typically cycled 40-45 times between -0.5 V and +0.5 V (*vs.* Ag/AgCl) to obtain fully reduced and oxidized state of *Pa*-Cti, respectively. The equilibration time after the potential change was 5 min and for each state, two spectra (256 scans and 4 cm^{-1} resolution) were recorded and averaged. Infrared spectra were humidity and baseline corrected.

UV-Visible redox titration

UV-Visible titrations were carried out in the same thin-layer cell as described above for redox induced infrared spectroscopy. The spectra were recorded at 10 °C with a Cary 300 spectrometer coupled to a potentiostat. The step of the potential shift was 50 mV in the regions far from the redox transition and 25mV in the redox transition region. The system equilibration time was at least 30 min and the potential was changed when the spectra did not evolve anymore. Each titration (oxidative and reductive) was performed at least twice, the spectral resolution was 1 nm. All the titration curves are presented *vs.* the standard hydrogen electrode (SHE).

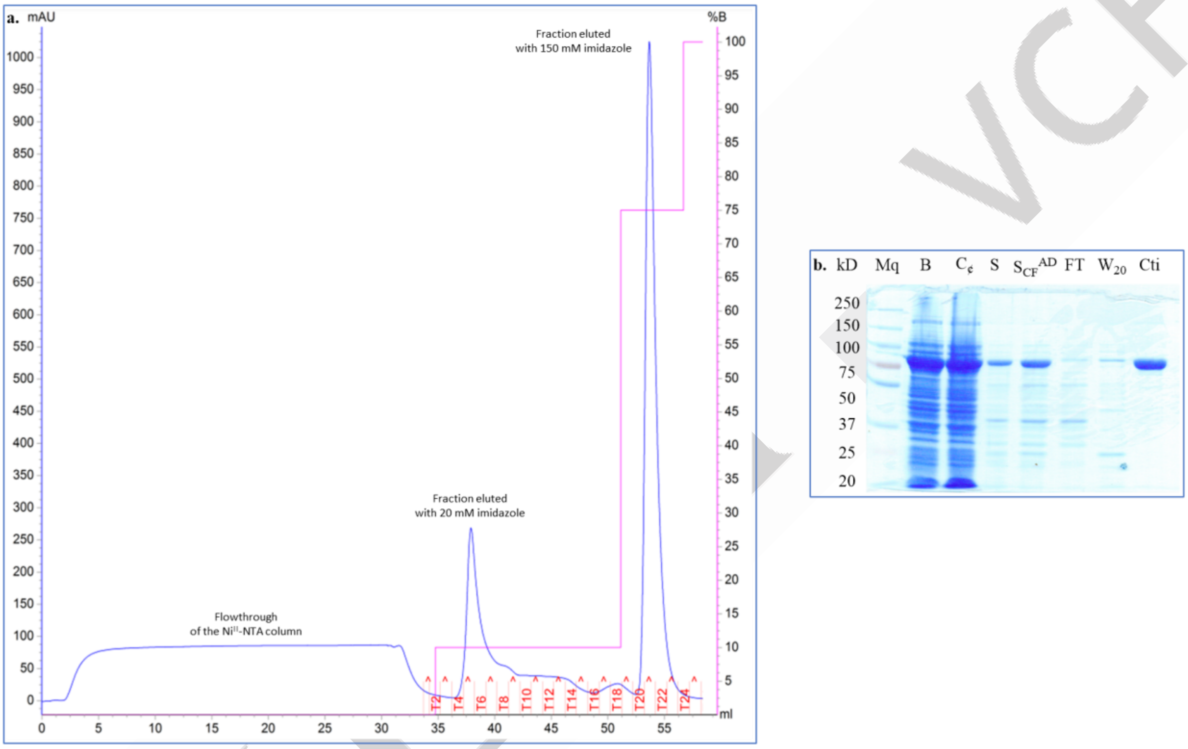

Figure S1. Analysis of *Pa*-Cti production expressed in *Escherichia coli* BL21(DE3) using the pET22b vector containing the *cti* gene and in the presence of the pEC86 vector containing the *cytochrome*-c *maturation* genes. a. Elution Profile. The blue line represents the eluate absorbance measured at 280 nm. The pink line represents the concentration gradient (buffer A: 50 mM Na_2HPO_4, 300 mM NaCl pH 8; buffer B: 50 mM Na_2HPO_4, 300 mM NaCl, 200 mM imidazole, pH 8). b. 10% SDS-PAGE Gel. Mq: molecular weight marker; B: bacterial pellet; C¢: spheroplast pellet; S: supernatant; S_{CF}^{AD}: concentrated and filtered supernatant after dialysis; FT: flowthrough of the Ni^{II}-NTA column; W_{20}: fraction eluted with 20 mM imidazole in buffer A; Cti: purified Pa-Cti.

WILEY-VCH

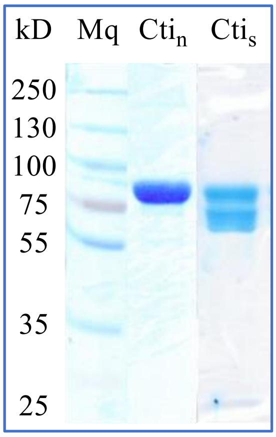

Figure S2. 10% SDS-PAGE analysis of *Pseudomonas aeruginosa* Cti production in the presence of *cytochrome*-c *maturation* genes. Mq: molecular weight marker; Cti$_n$: Cti purified when cell lysis was performed by osmotic shock; Cti$_s$: Cti purified when cell lysis was performed by sonication.

WILEY-VCH

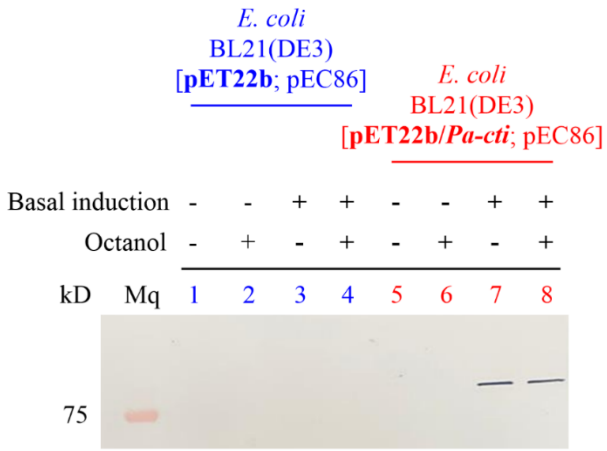

Figure S3. Western blot analysis of basal *Pa*-Cti expression after induction of the *cis-trans* isomerization reaction of UFA by octanol. Molecular weight marker (Mq); bacterial pellet of *Escherichia coli* BL21(DE3)[pEC86] cells transformed with pET22b plasmid (1 – 4) or with pET22b/*Pa-cti* plasmid (5 – 8). Basal induction of *Pa*-Cti was achieved by adding 10 µM IPTG (3, 4, 7, and 8). Stress was initiated by adding octanol to reach 2.5 mM (2, 4, 6, and 8).

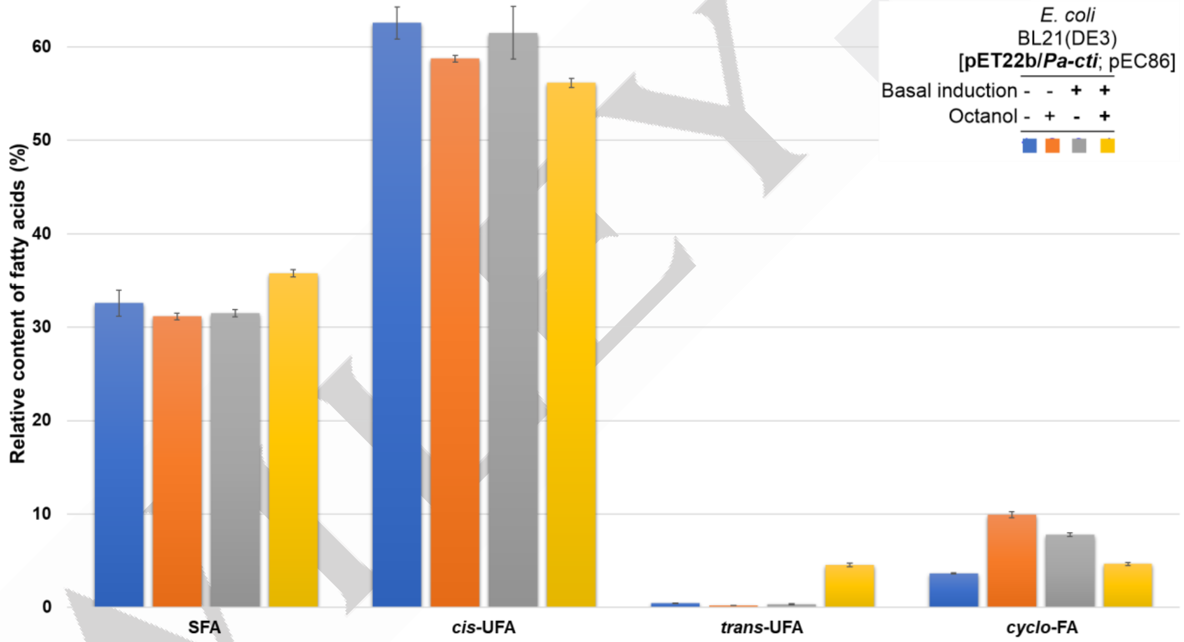

Figure S4. Distribution of membrane fatty acids in *Escherichia coli* BL21(DE3)[pET22b/*Pa-cti*; pEC86] cultured at 30 °C. *Escherichia coli* BL21(DE3)[pEC86] cells were transformed with the pET22b/*Pa-cti* plasmid (■,■,■,■). Basal induction of the Cti protein was achieved by adding 10 µM IPTG (■,■). Stress was initiated by adding octanol to a final concentration of 2.5 mM (■).

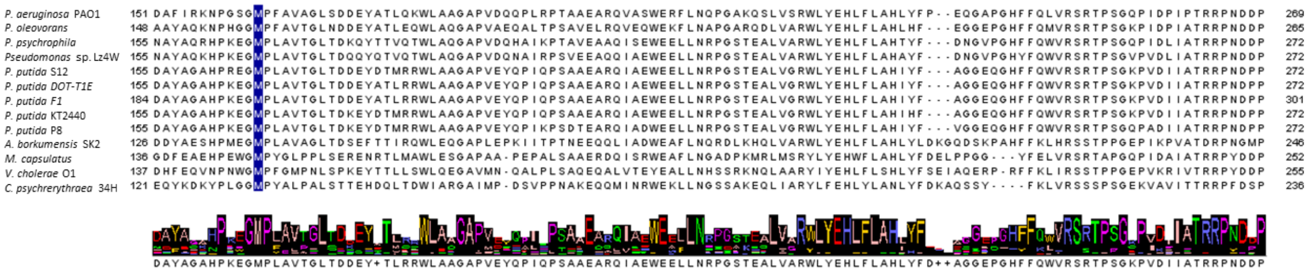

Figure S5. Multiple sequence alignment of the Cti proteins. This figure displays the sequence alignment of Cti from *Pseudomonas aeruginosa* PAO1 (NP_250537), *Pseudomonas oleovorans* (KJU78609), *Pseudomonas psychrophila* (*Pseudomonas* sp. E-3) (BAB41104), *Pseudomonas sp*. Lz4W (*Pseudomonas syringae* Lz4W) (CAD59690), *Pseudomonas putida* S12 (AJA16735), *Pseudomonas putida* DOT-T1E (AAD41252), *Pseudomonas putida* F1 (ABQ79445), *Pseudomonas putida* KT2440 (AAN67989), *Pseudomonas putida* P8 (CAA04438), *Alcanivorax borkumensis* SK2 (CAL17148), *Methylococcus capsulatus* str. Bath (AAU92135), *Vibrio cholerae* O1 biovar El Tor str. N16961 (AWA80188) and *Colwellia psychrerythraea* 34H (AAZ24945). The conserved methionine is shown in blue.

Table S1. BLAST analysis based on the Cti protein sequence of *Pseudomonas aeruginosa* PAO1.

Microorganisms	Identity (%)	Reference NCBI
***Pseudomonas aeruginosa* PAO1**	100	NP_250537
Pseudomonas oleovorans	70.95	KJU78609
***Pseudomonas psychrophila* (*Pseudomonas* sp. E-3)[a]**	67.21	BAB41104
***Pseudomonas* sp. Lz4W (*Pseudomonas syringae* Lz4W)[a]**	67.07	CAD59690
***Pseudomonas putida* S12**	65.45	AJA16735
***Pseudomonas putida* DOT-T1E**	65.32	AAD41252
***Pseudomonas putida* F1**	65.32	ABQ79445
***Pseudomonas putida* KT2440**	64.80	AAN67989
***Pseudomonas putida* P8**	64.80	CAA04438
***Alcanivorax borkumensis* SK2**	52.57	CAL17148
***Methylococcus capsulatus* str. Bath**	38.45	AAU92135
***Vibrio cholerae* O1 biovar El Tor str. N16961**	38.78	AWA80188
***Colwellia psychrerythraea* 34H**	36.04	AAZ24945

[a] Fomer name.

WILEY-VCH

WILEY-VCH

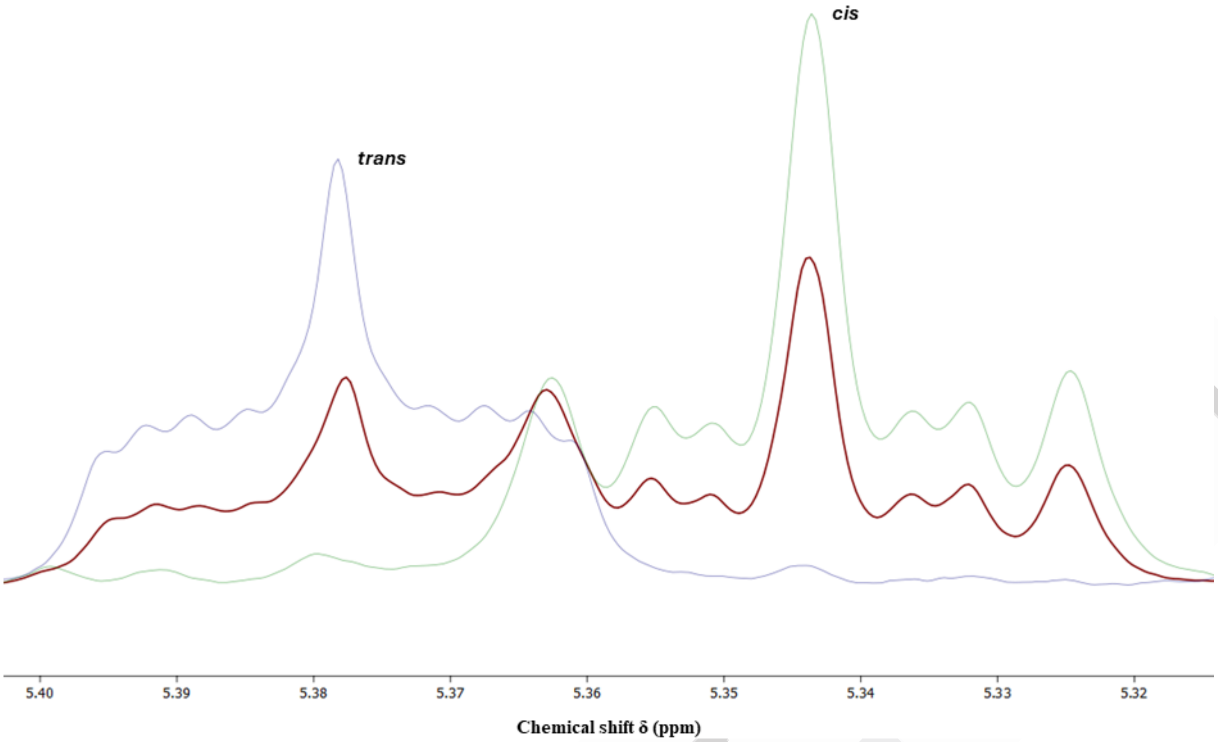

Figure S6. ^{1}H NMR spectra (300 MHz) of FAME obtained after incubation of *Pa*-Cti WT or the mutants *Pa*-Cti M163A and *Pa*-Cti M163H with palmitoleic acid (16:1 cis-Δ^{9}) as substrate. Palmitoleic acid (16:1 *cis*-Δ^{9}, 1 mM) was incubated at 30 °C with WT *Pa*-Cti, *Pa*-Cti M163A or *Pa*-Cti M163H (0.3 µM) in 50 mM Na_2HPO_4, 300 mM NaCl, pH 8 for 18 h. The reaction was quenched using 6N HCl/MeOH. FAME were analyzed by ^{1}H NMR (300 MHz) after 1 h heating at 90 °C followed by extraction with CH_2Cl_2. Tests were conducted with WT *Pa*-Cti (purple), *Pa*-Cti M163A (brown), and *Pa*-Cti M163H (green). Only the 5.3-5.4 ppm region corresponding to the vinyl protons of the cis and trans isomers of 16:1 FAME are represented.

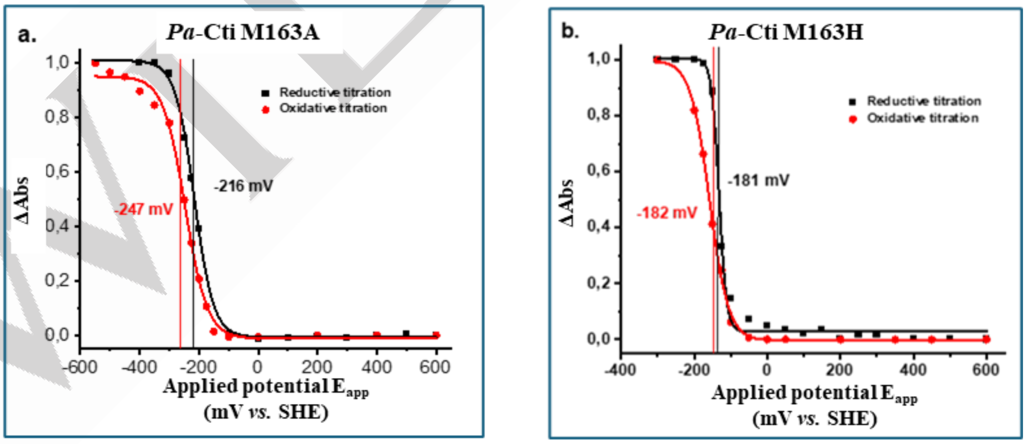

Figure S7. Determination of the average redox potential (E_m) for the mutants *Pa*-Cti M163A and *Pa*-Cti M163H. a. *Pa*-Cti M163A; b. *Pa*-Cti M163H. Each mutant (600 µM) in K_2HPO_4 buffer at pH 8 was loaded into a thin-layer electrochemical cell. The graph ΔAbs vs. E_{app} was constructed using the α band (556 nm). Curve fitting of the titration curves was performed based on the Nernst equation with n = 1 to determine the redox potential.

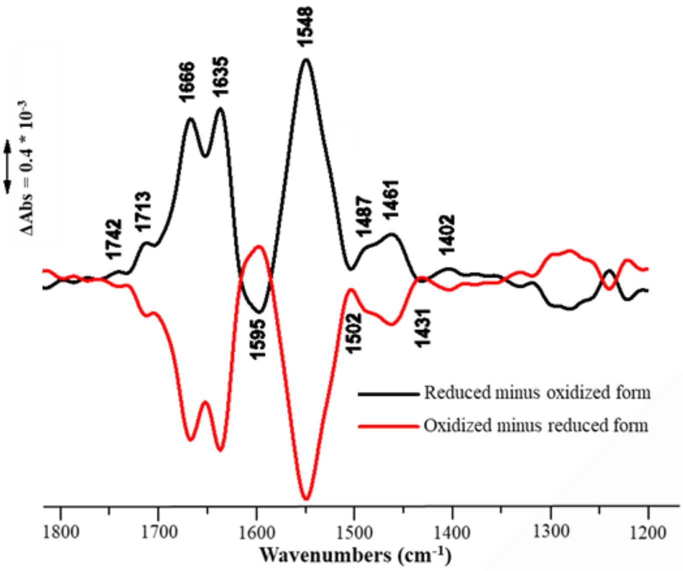

Figure S8. Differential infrared spectra of *Pa*-Cti. The analysis of *Pa*- Cti (350 µM) in K_2HPO_4 pH 8 buffer was conducted using a thin-layer electrochemical cell.

Table S2. Tentative assignment of the observed bands in the differential infrared spectrum of the reduced form minus the oxidized form of *Pa*-Cti.

Wavenumbers (cm^{-1}) +	Wavenumbers (cm^{-1}) -	Attempted assignment
1742		ν(C=O) Asp/Glu in the protonated state
1713		ν(C=O) Asp/Glu in the protonated state
1666		$\nu(CN_3H_5)_{as}$ Arg<br>Amide I (turns, α helix)
1635		$\nu(CN_3H_5)_s$ Arg<br>Amide I (β sheet)
	1595	ν_{37} ($C_\beta C_\beta$) heme-*b*/*c*
1548		Amide II<br>ν_{38} ($C_\alpha C_m$) heme-*b*/*c*<br>$\nu(COO^-)_{as}$ Asp/Glu in the deprotonated state<br>$\nu(COO^-)_{as}$ heme propionates in the deprotonated state
	1502	Vibration of the phenolic cycle Tyr
1487		ν_{39} ($C_\alpha C_m$) heme-*b*/*c*
1461		ν_{39} (CβCβ) heme-*b*/*c*
	1431	$\nu(COO^-)_s$ Asp/Glu in the deprotonated state
1402		$\nu(COO^-)_s$ Asp/Glu in the deprotonated state<br>$\nu(COO^-)_s$ heme propionates in the deprotonated state

The major contributions from the peptide backbone are observed in the spectral range between 1620 cm^{-1} and 1680 cm^{-1}, corresponding to the Amide I band.[7] Signals originating from β-sheets can be identified between 1680 – 1690 cm^{-1} and 1620 – 1645 cm^{-1}, while those associated with α-helices are predominantly located between 1650 – 1670 cm^{-1}. Signals characteristic of the Amide II band are also expected to appear between 1520 cm^{-1} and 1570 cm^{-1}, reflecting the coupling of ν(C—N) and δ(N—H) vibrations. Vibrations typical of heme proteins manifest in the range of 1500 cm^{-1} to 1620 cm^{-1}. The ν_{37} ($C_\beta C_\beta$) bands can be observed between 1560 – 1620 cm^{-1}, while the ν_{38} ($C_\alpha C_m$) bands fall within the range of 1535 – 1570 cm^{-1}. A plausible position for the latter is around 1548 cm^{-1} for *Pa*-Cti. Nevertheless, this specific band can be challenging to attribute due to the presence of other spectral contributions in this region. Bands corresponding to the propionates of hemes in their protonated form are typically found between 1710 – 1675 cm^{-1}, whereas in their deprotonated form, they are observed between 1530 – 1570 cm^{-1} for the $\nu(COO^-)_{as}$ vibrations and around 1400 cm^{-1} for the $\nu(COO^-)_s$ vibrations. In *Pa*-Cti, these signals were detected at 1402 cm^{-1} and 1548 cm^{-1}. Arginine residues exhibit vibrational modes $\nu(CN_3H_5)_{as}$ at approximately 1675 cm^{-1} and $\nu(CN_3H_5)_s$ around 1635 cm^{-1}.[8] In our study, an arginine residue may contribute to vibrations at 1666 cm^{-1} and 1635 cm^{-1}. Aspartic and glutamic acids possess ν(C=O) vibrational modes in their protonated state, typically appearing in the range of 1710 – 1790 cm^{-1}, while their deprotonated state is characterized by $\nu(COO^-)_{as}$ vibrations between 1530 – 1590 cm^{-1} and $\nu(COO\text{-})_s$ vibrations between 1380 – 1430 cm^{-1}.[8] The deprotonated forms (1402, 1431, 1548 cm^{-1}) and protonated forms (1713, 1742 cm^{-1}) of aspartic or glutamic acids might be observable in the differential IR spectrum of *Pa*-Cti. Tyrosine bands can also be discerned in their protonated form around 1520 cm^{-1} and between 1235 – 1270 cm^{-1}, whereas deprotonated forms are typically observed around 1270 – 1500 cm^{-1}.[8]

Table S3. Plasmids.

Plasmid	Features and uses	Source
**pET22b(+)**	Amp^r, expression vector containing the pelB signal sequence, the polyhistidine sequence (His_6-tag), and the T7 promoter.	**Novagen**
**pET22b(+)/*Pa-cti***	Amp^r, derived from pET22b(+) containing the coding sequence of pelB and Cti from *P. aeruginosa* (pelB SP-Cti-His_6).	**This study**
**pET22b(+)/*Pa-cti* M163A**	Amp^r, derived from pET22b(+)/*Pa-cti* containing the M163A mutation.	**This study**
**pET22b(+)/*Pa-cti* M163H**	Amp^r, derived from pET22b(+)/*Pa-cti* containing the M163H mutation.	**This study**
**pET30b(+)**	Kan^r, expression vector containing the polyhistidine sequence (His_6-tag) and the T7 promoter.	**Novagen**
**pET30b(+)/*Pa-cti*[a]**	Kan^r, derived from pET30b(+), it contains the coding sequence of Cti from *P. aeruginosa* (native signal peptide-Cti-His_6).	**Genescript**
**pEC86**	Cat^r, Expression vector containing the maturation genes (*ccm*) from *E. coli* under the control of the *tet* promoter of pACY184.	**CCOS[b]**

The plasmids are stored at -20 °C in 10 mM Tris-HCl at pH 8.5. The gene encoding the enzyme Cti from *P. aeruginosa* was synthesized after codon optimization by the company Genscript and then cloned into the pET30b vector. Amp^r: ampicillin resistance; Cat^r: chloramphenicol resistance; CCOS: Culture Collection of Switzerland; Kan^r: kanamycin resistance; SP: signal peptide.

Table S4. Oligonucleotides.

Oligonucleotides	Sequence (5’-...-3’)	Features and uses
**For/*Pa-cti* without SP**	TTT**C·CATG**GCGGAAATTAGCTACA	PCR amplification of the *cti gene* incorporating an NcoI restriction site (**C·CATG**)
**Rev/*Pa-cti* without SP**	GA**C·CTCGAG**GTTCTCGTAACGGT	PCR amplification of the *cti gene* incorporating an XhoI restriction site (**C·CTCGAG**)
**For/*Pa-cti* M163A**	CCCGGGTAGCGGC**GCG**CCGTTTGCGGTT	PCR amplification of the sense strand of the *cti gene* incorporating the M163A mutation
**Rev/*Pa-cti* M163A**	AACCGCAAACGG**CGC**GCCGCTACCCGGG	PCR amplification of the antisense strand of the *cti gene* incorporating the M163A mutation
**For/*Pa-cti* M163H**	AACCCGGGTAGCGGC**CAT**CCGTTTGCGGTTGCG	PCR amplification of the sense strand of the *cti gene* incorporating the M163H mutation
**Rev/*Pa-cti* M163H**	CGCAACCGCAAACGG**ATG**GCCGCTACCCGGGTT	PCR amplification of the antisense strand of the *cti gene* incorporating the M163H mutation

Oligonucleotides are stored at -20°C in sterilized ultrapure water. Primers used to construct M/A(H) mutants with the codon introduced in the mutant shown in bold. SP: signal peptide.

WILEY-VCH

(a.)
**ATG CTG CCG CGT CCG CTG GTT GGT CTG GCG TTC GTT CTG AGC AGC TTT ATC CAG AGC GTG AGC GCG GCG GAA ATT AGC
TAC A**GC CGT GAT GTG CAA CCG ATC TTC ACC GCG AAG TGC GTT GCG TGC CAC GCG TGC TAT GAC AGC CCG TGC CAG CTG AAC
CTG AGC AGC GCG GAA GGT GCG CAA CGT GGC GCG AAC CAA CTG CCG GTG TAC GAC GGT ACC CGT ACC AAG GCG CAA GAA ACC
ACC CGT CTG TAT CTG GAT GCG CAC GGC GCG GAT GCG TGG CGT CGT AAA GAC TTT TGG AGC GTT CTG GAA CCG CAG GAT GGT
CAA GCG GCG CTG ATG GCG CGT ATG CTG GAG CTG GGT CAC AGC CAG CCG CTG CAA CCG AAC GCG AAG ATT CCG GAA GGT CTG
GAC ATC AGC ATT AAC CGT GCG AAC CAA TGC CCG ACC CCG GCG AGC ATT GAT GCG TTC ATT CGT AAG AAC CCG GGT AGC GGC
ATG CCG TTT GCG GTT GCG GGT CTG AGC GAC GAT GAG TAC GCG ACC CTG CAG AAA TGG CTG GCG GCG GGT GCG CCG GTG GAT
CAG CAG CCG CTG CGT CCG ACC GCG GCG GAG GCG CGT CAA GTT GCG AGC TGG GAA CGT TTC CTG AAC CAG AGC GGT GCG AAA
CAA AGC CTG GTG AGC CGT TGG CTG TAC GAA CAC CTG TTC CTG GCG CAC CTG TAT TTT CCG GAG CAG GGT GCG CCG GGC CAC
TTC TTT CAA CTG GTT CGT AGC CGT ACC CCG AGC GGT CAG CCG ATT GAC CCG ATT CCG ACC CGT CGT CCG AAC GAC GAT CCG
GGT AAC AGC TTT TAC TAT CGT CTG TGG CCG ATC CAA GGC GTG ATT GTT CAC AAG ACC CAC ATC ACC TAC CCG CTG ACC GCG
AAG AAA CTG GAG CGT GTG CAG GAA CTG TTC TTT GGT ACC CAA TGG AAC ACC GAT AAA GTG CCG GGT TAT GGC GTT CAG AGC
CGT GCG AAC CCG TTC GTG ACC TTT GCG GCG ATC CCG CCG CGT GCG CGT TAC CAA TTC ATG CTG GAC AAC GCG GAA TAT TTC
ACC CGT ACC TTT ATT CGT GGT CCG GTG TGC CGT GGT CAG ATT GCG ACC GAT GTT ATT CGT GAC AAC TTC TGG GTG GTT TTT CAG
GAC CCG GAG CAA GAC CTG TTC GTG ACC GAC GCG AAC TTT CGT GCG CAA AGC GAA CCG CTG CTG GCG CTG CCG GGT CAA ATC
GAC GAG CTG AAG AAC CTG CTG GGC CTG TGG AGC GCG TAC CGT GAT AAA CGT AAC GAG TAT GAA GAT CTG CGT CAG GAC GTT
TAT GCG GAT GCT CCG CCG CCG ACC TGG AAC ACC ATC TGG CAC GGT AAC GAC AAC GCG CTG CTG AGC ATT TTC CGT CAG TTT
GAT AGC GCG AGC GTG CGT AAG GGT CTG CTG GGC GAA GTT CCG CAA ACC CTG TGG CTG ATG GAC TAC CCG CTG TTC GAG CGT
ACC TAC TAT GGT CTG GTG GTT AAC TTC GAT GTG TTT GGC AAC GTT AGC CAC CAG GCG CAA ACC CGT CTG TAT TTC GAC CTG ATT
CGT AAC GGT GCG GAA CAG AAC TTT CTG CGT CTG ATG CCG GTG GAT GCG CGT CAG CCG CTG CTG GAC GAT TGG TAC CAA AAC
AGC GGC AAG CTG AAA ATG TGG ATG GAC TAT CAG GCG TTC GAC GAT GAT ACC CCG AGC GCG CTG GGT CTG CCG GAG AAA CAA
CCG AAG AAA GCG TTT GCG GAG GAA CTG CTG CGT CGT TAC GGC GAC CTG AAC GCG CGT CCG GAC CCG ATC AAC CGT TGC CTG
GAT GGT AAC TGC TAT CGT CCG GGC ATT GAC CGT GAG CTG CAG GAT GCG GAA CAA GCG TTC AGC CGT CTG GTG AGC CGT CCG
GCG GCG GGT CTG AAG GTT ATC GAG CGT TTT CCG GAA GCG ACC ATG CTG CGT ATC CGT ACC AGC AGC GGC AAA CGT GAA ATT
TAC ACC GTG CTG CGT AAC CGT GCG CAC AGC AAC GTT GCG TTC ATG CTG GGC GAG AGC CTG CGT TAT CAG CCG GGC CTG GAC
ACC CTG ACC ATT TAC CCG GGT GTG CTG AGC AGC TAT CCG AAC TTC ATG TTT GAC CTG CCG GCG ACC GAT GCG GAA GCG TTC
GTT GGT GCG CTG GAG GCG GCG AAG AGC AGC GAA GAT TTT GAC AAA GTG GTT GAG CGT TGG GGC GTG CGT CGT AGC AAC CCG
CAG TTC TGG AGC TAC TTT CAC GAT CTG GAG GCG TAT ATC CGT GAA ACC GAA CCG GTT GAA GCG GGC GCG CTG GAC ATG A**AC
CGT TAC GAG AAC CTC GAG** **CAC CAC CAC CAC CAC CAC**

(b.)
M L P R P L V G L A F V L S S F I Q S V S A A E I S Y S R D V Q P I F T A K C V A C H A C Y D S P C Q L N L S S A E G A Q R G A N Q L P V
Y D G T R T K A Q E T T R L Y L D A H G A D A W R R K D F W S V L E P Q D G Q A A L M A R M L E L G H S Q P L Q P N A K I P E G L D I S
I N R A N Q C P T P A S I D A F I R K N P G S G M P F A V A G L S D D E Y A T L Q K W L A A G A P V D Q Q P L R P T A A E A R Q V A S W E
R F L N Q S G A K Q S L V S R W L Y E H L F L A H L Y F P E Q G A P G H F F Q L V R S R T P S G Q P I D P I P T R R P N D D P G N S F Y Y R
L W P I Q G V I V H K T H I T Y P L T A K K L E R V Q E L F F G T Q W N T D K V P G Y G V Q S R A N P F V T F A A I P P R A R Y Q F M L D
N A E Y F T R T F I R G P V C R G Q I A T D V I R D N F W V V F Q D P E Q D L F V T D A N F R A Q S E P L L A L P G Q I D E L K N L L G L W
S A Y R D K R N E Y E D L R Q D V Y A D A P P P T W N T I W H G N D N A L L S I F R Q F D S A S V R K G L L G E V P Q T L W L M D Y P L
F E R T Y Y G L V V N F D V F G N V S H Q A Q T R L Y F D L I R N G A E Q N F L R L M P V D A R Q P L L D D W Y Q N S G K L K M W M D
Y Q A F D D D T P S A L G L P E K Q P K K A F A E E L L R R Y G D L N A R P D P I N R C L D G N C Y R P G I D R E L Q D A E Q A F S R L V S
R P A A G L K V I E R F P E A T M L R I R T S S G K R E I Y T V L R N R A H S N V A F M L G E S L R Y Q P G L D T L T I Y P G V L S S Y P N F
M F D L P A T D A E A F V G A L E A A K S S E D F D K V V E R W G V R R S N P Q F W S Y F H D L E A Y I R E T E P V E A G A L D M N **R Y
E N L E** **H H H H H H**

Figure S9. (a.) Coding sequence and (b.) deduced protein sequence of Cti from *Pseudomonas aeruginosa* in the pET30b expression vector. The coding sequence and deduced protein sequence of the native *Pseudomonas aeruginosa* Cti signal peptide and polyhistidine tag (His_6-tag) are shown in blue and purple respectively. The primer pair used to amplify the cti gene encoding the protein form without signal peptide by PCR are shown in bold and underlined.

WILEY-VCH

(a.)

ATG AAA TAC CTG CTG CCG ACC GCT GCT GCT GGT CTG CTG CTC CTC GCT GCC CAG CCG GCG ATG GCC ATG GCG GAA ATT
AGC TAC AGC CGT GAT GTG CAA CCG ATC TTC ACC GCG AAG **TGC GTT GCG TGC CAC** GCG TGC TAT GAC AGC CCG TGC CAG CTG
AAC CTG AGC AGC GCG GAA GGT GCG CAA CGT GGC GCG AAC CAA CTG CCG GTG TAC GAC GGT ACC CGT ACC AAG GCG CAA GAA
ACC ACC CGT CTG TAT CTG GAT GCG CAC GGC GCG GAT GCG TGG CGT CGT AAA GAC TTT TGG AGC GTT CTG GAA CCG CAG GAT
GGT CAA GCG GCG CTG ATG GCG CGT ATG CTG GAG CTG GGT CAC AGC CAG CCG CTG CAA CCG AAC GCG AAG ATT CCG GAA GGT
CTG GAC ATC AGC ATT AAC CGT GCG AAC CAA TGC CCG ACC CCG GCG AGC ATT GAT GCG TTC ATT CGT AAG AAC CCG GGT AGC
GGC **ATG** CCG TTT GCG GTT GCG GGT CTG AGC GAC GAT GAG TAC GCG ACC CTG CAG AAA TGG CTG GCG GCG GGT GCG CCG GTG
GAT CAG CAG CCG CTG CGT CCG ACC GCG GCG GAG GCG CGT CAA GTT GCG AGC TGG GAA CGT TTC CTG AAC CAG AGC GGT GCG
AAA CAA AGC CTG GTG AGC CGT TGG CTG TAC GAA CAC CTG TTC CTG GCG CAC CTG TAT TTT CCG GAG CAG GGT GCG CCG GGC
CAC TTC TTT CAA CTG GTT CGT AGC CGT ACC CCG AGC GGT CAG CCG ATT GAC CCG ATT CCG ACC CGT CGT CCG AAC GAC GAT
CCG GGT AAC AGC TTT TAC TAT CGT CTG TGG CCG ATC CAA GGC GTG ATT GTT CAC AAG ACC CAC ATC ACC TAC CCG CTG ACC
GCG AAG AAA CTG GAG CGT GTG CAG GAA CTG TTC TTT GGT ACC CAA TGG AAC ACC GAT AAA GTG CCG GGT TAT GGC GTT CAG
AGC CGT GCG AAC CCG TTC GTG ACC TTT GCG GCG ATC CCG CCG CGT GCG CGT TAC CAA TTC ATG CTG GAC AAC GCG GAA TAT
TTC ACC CGT ACC TTT ATT CGT GGT CCG GTG TGC CGT GGT CAG ATT GCG ACC GAT GTT ATT CGT GAC AAC TTC TGG GTG GTT TTT
CAG GAC CCG GAG CAA GAC CTG TTC GTG ACC GAC GCG AAC TTT CGT GCG CAA AGC GAA CCG CTG CTG GCG CTG CCG GGT CAA
ATC GAC GAG CTG AAG AAC CTG CTG GGC CTG TGG AGC GCG TAC CGT GAT AAA CGT AAC GAG TAT GAA GAT CTG CGT CAG GAC
GTT TAT GCG GAT GCT CCG CCG CCG ACC TGG AAC ACC ATC TGG CAC GGT AAC GAC AAC GCG CTG CTG AGC ATT TTC CGT CAG
TTT GAT AGC GCG AGC GTG CGT AAG GGT CTG CTG GGC GAA GTT CCG CAA ACC CTG TGG CTG ATG GAC TAC CCG CTG TTC GAG
CGT ACC TAC TAT GGT CTG GTG GTT AAC TTC GAT GTG TTT GGC AAC GTT AGC CAC CAG GCG CAA ACC CGT CTG TAT TTC GAC
CTG ATT CGT AAC GGT GCG GAA CAG AAC TTT CTG CGT CTG ATG CCG GTG GAT GCG CGT CAG CCG CTG CTG GAC GAT TGG TAC
CAA AAC AGC GGC AAG CTG AAA ATG TGG ATG GAC TAT CAG GCG TTC GAC GAT GAT ACC CCG AGC GCG CTG GGT CTG CCG GAG
AAA CAA CCG AAG AAA GCG TTT GCG GAG GAA CTG CTG CGT CGT TAC GGC GAC CTG AAC GCG CGT CCG GAC CCG ATC AAC CGT
TGC CTG GAT GGT AAC TGC TAT CGT CCG GGC ATT GAC CGT GAG CTG CAG GAT GCG GAA CAA GCG TTC AGC CGT CTG GTG AGC
CGT CCG GCG GCG GGT CTG AAG GTT ATC GAG CGT TTT CCG GAA GCG ACC ATG CTG CGT ATC CGT ACC AGC AGC GGC AAA CGT
GAA ATT TAC ACC GTG CTG CGT AAC CGT GCG CAC AGC AAC GTT GCG TTC ATG CTG GGC GAG AGC CTG CGT TAT CAG CCG GGC
CTG GAC ACC CTG ACC ATT TAC CCG GGT GTG CTG AGC AGC TAT CCG AAC TTC ATG TTT GAC CTG CCG GCG ACC GAT GCG GAA
GCG TTC GTT GGT GCG CTG GAG GCG GCG AAG AGC AGC GAA GAT TTT GAC AAA GTG GTT GAG CGT TGG GGC GTG CGT CGT AGC
AAC CCG CAG TTC TGG AGC TAC TTT CAC GAT CTG GAG GCG TAT ATC CGT GAA ACC GAA CCG GTT GAA GCG GGC GCG CTG GAC
ATG AAC CGT TAC GAG AAC CTC GAG **CAC CAC CAC CAC CAC CAC**

(b.)

M K Y L L P T A A A G L L L L A A Q P A M A M A E I S Y S R D V Q P I F T A K **C V A C H** A C Y D S P C Q L N L S S A E G A Q R G A N Q
L P V Y D G T R T K A Q E T T R L Y L D A H G A D A W R R K D F W S V L E P Q D G Q A A L M A R M L E L G H S Q P L Q P N A K I P E G L
D I S I N R A N Q C P T P A S I D A F I R K N P G S G **M** P F A V A G L S D D E Y A T L Q K W L A A G A P V D Q Q P L R P T A A E A R Q V A
S W E R F L N Q S G A K Q S L V S R W L Y E H L F L A H L Y F P E Q G A P G H F F Q L V R S R T P S G Q P I D P I P T R R P N D D P G N S F
Y Y R L W P I Q G V I V H K T H I T Y P L T A K K L E R V Q E L F F G T Q W N T D K V P G Y G V Q S R A N P F V T F A A I P P R A R Y Q F
M L D N A E Y F T R T F I R G P V C R G Q I A T D V I R D N F W V V F Q D P E Q D L F V T D A N F R A Q S E P L L A L P G Q I D E L K N L L
G L W S A Y R D K R N E Y E D L R Q D V Y A D A P P P T W N T I W H G N D N A L L S I F R Q F D S A S V R K G L L G E V P Q T L W L M D
Y P L F E R T Y Y G L V V N F D V F G N V S H Q A Q T R L Y F D L I R N G A E Q N F L R L M P V D A R Q P L L D D W Y Q N S G K L K M W
M D Y Q A F D D D T P S A L G L P E K Q P K K A F A E E L L R R Y G D L N A R P D P I N R C L D G N C Y R P G I D R E L Q D A E Q A F S R
L V S R P A A G L K V I E R F P E A T M L R I R T S S G K R E I Y T V L R N R A H S N V A F M L G E S L R Y Q P G L D T L T I Y P G V L S S Y
P N F M F D L P A T D A E A F V G A L E A A K S S E D F D K V V E R W G V R R S N P Q F W S Y F H D L E A Y I R E T E P V E A G A L D M
N R Y E N L E **H H H H H H**

Figure S10. (a.) Coding sequence and (b.) deduced protein sequence of Cti from *Pseudomonas aeruginosa* in the pET22b expression vector. The coding sequence and deduced protein sequence of the pelB signal peptide and polyhistidine tag (His_6-tag) are shown in dark blue and purple respectively. The heme binding motif is shown in red. Methionine mutated in the course of this work is shown in light blue. Due to the plasmid construction, a one-amino acid shift (+1) is present in the peptide sequence of *Pa*-Cti after expression of the *cti* gene from *P. aeruginosa* in plasmid pET22b(+)/*Pa-cti*. A methionine after the pelB signal peptide is present in the recombinant Cti protein (native *P. aeruginosa* Cti: VSA-AEI; recombinant *P. aeruginosa* Cti: AMA-MAEI). The cleavage site of the signal peptides was predicted using SignalP 6.0.[9]

WILEY-VCH

References

[1] M. M. Bradford, *Anal. Biochem.* **1976**, *72*, 248–254.
[2] L. Vendel Nielsen, T. P. Krogager, C. Young, C. Ferreri, C. Chatgilialoglu, O. Nørregaard Jensen, J. J. Enghild, *PLoS One* **2013**, *8*, e74283.
[3] N. Aldai, B. E. Murray, A. I. Nájera, D. J. Troy, K. Osoro, *J. Sci. Food Agric.* **2005**, *85*, 1073–1083.
[4] B. Tse Sum Bui, D. Florentin, A. Marquet, R. Benda, A. X. Trautwein, *FEBS Lett.* **1999**, *459*, 411–414.
[5] H. P. Gunnlaugsson, *Hyperfine Interact.* **2016**, *237*, 79–84.
[6] D. Moss, E. Nabedryk, J. Breton, W. Mäntele, *Eur. J. Biochem.* **1990**, *187*, 565–572.
[7] A. Barth, *Prog. Biophys. Mol. Biol.* **2000**, *74*, 141–173.
[8] M. Wolpert, P. Hellwig, *Spectrochim. Acta A Mol. Biomol. Spectrosc.* **2006**, *64*, 987–1001.
[9] F. Teufel, J. J. Almagro Armenteros, A. R. Johansen, M. H. Gíslason, S. I. Pihl, K. D. Tsirigos, O. Winther, S. Brunak, G. von Heijne, H. Nielsen, *Nat. Biotechnol.* **2022**, *40*, 1023–1025.

WILEY-VCH

WILEY-VCH
